# MAGEA6 Engages a YY1‐Dependent Transcription to Dictate Perineural Invasion in Colorectal Cancer

**DOI:** 10.1002/advs.202501119

**Published:** 2025-03-27

**Authors:** Hao Wang, Kexin He, Ruixue Huo, Weihan Li, Shan Zhang, Lu‐Ju Jiang, Hao Wu, Minhao Yu, Shu‐Heng Jiang, Junli Xue

**Affiliations:** ^1^ Department of Oncology Shanghai East Hospital School of Medicine Tongji University Shanghai 200092 P. R. China; ^2^ Department of Gastrointestinal Surgery Ren Ji Hospital School of Medicine Shanghai Jiao Tong University Shanghai 200127 P. R. China; ^3^ State Key Laboratory of Systems Medicine for Cancer Shanghai Cancer Institute Ren Ji Hospital School of Medicine Shanghai Jiao Tong University Shanghai 200240 P. R. China

**Keywords:** cancer neuroscience, melanoma‐associated antigen a6, perineural invasion, schwann cell, yin‐yang 1

## Abstract

Perineural invasion (PNI), characterized by tumor cells surrounding and invading nerves, is associated with poor prognosis in colorectal cancer (CRC). Understanding the mechanisms of PNI is crucial for developing targeted therapies to impede tumor progression. In this study, clinical information and transcriptome data are obtained from the TCGA database. Stable *MAGEA6* knockdown CRC cell lines are established to investigate the impact of MAGEA6 on CRC malignancy. Immunohistochemical staining is used to assess the clinical significance of MAGEA6. Rectal orthotopic and sciatic nerve invasion models are employed to verify the role of MAGEA6 in PNI. Schwann cells (SCs) infiltration and recruitment by CRC cells are assessed using ssGSEA and co‐culture experiments. The results reveal that MAGEA6 is a key regulator of PNI, with its expression correlating with poor prognosis. *MAGEA6* knockdown reduces CRC cell migration, invasion, and PNI ability. Moreover, CRC cells recruit SCs, with CXCL1 promoting SCs migration. Mechanistically, MAGEA6 inhibits YY1 ubiquitination, stabilizing YY1 expression and enhancing SC recruitment via YY1‐mediated *CXCL1* transcription. These findings suggest that MAGEA6 enhances CRC invasiveness and PNI by stabilizing YY1, which upregulates CXCL1 secretion and promotes SC recruitment. This interaction underscores the critical role of MAGEA6 in PNI and highlights a potential therapeutic target in CRC.

## Graphical abstract

This study investigates the role of MAGEA6 in perineural invasion (PNI) in colorectal cancer (CRC). MAGEA6 promotes CRC invasiveness by inhibiting YY1 ubiquitination, enhancing CXCL1 secretion, and recruiting Schwann cells. These findings highlight the potential of targeting the MAGEA6/YY1/CXCL1 axis for therapeutic strategies against PNI and tumor progression in CRC.

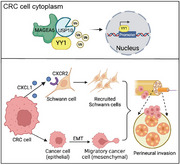

## 1 Introduction

Nerves play an important role in the development and progression of tumors. The concept of nerve involvement in cancer was first proposed in the 19th century by the pathologist Rudolf Virchow, who observed nerve infiltration in tumor tissues. Within the tumor microenvironment (TME), dynamic reciprocity exists where cancer cells secrete neurotrophic factors to induce axonogenesis and neuropathic pain,^[^ ^1^, ^2^, ^3^ ^]^ while neural components reciprocally release neurotransmitters and neuropeptides that potentiate tumor proliferation, angiogenesis, and immune evasion.^[^ ^4^, ^5^, ^6^, ^7^, ^8^ ^]^ This bidirectional crosstalk establishes a permissive niche for cancer progression.

Perineural invasion (PNI) refers to the infiltration of tumor cells into the perineural space, defined as tumor cells around nerves, involving over 33% of the nerve circumference or invasion into the nerve sheath.^[^ ^9^ ^]^ In the process of PNI, tumor cells can use the nerve fibers as leading strands to migrate to other sites, which is linked to increased tumor invasion and metastasis. Schwann cell (SC) is the myelin‐forming cell of the peripheral nervous system (PNS) and has been demonstrated to be a pivotal factor in PNI.^[^ ^10^ ^]^ A study on colon cancer revealed that SCs migrate toward tumor cells before PNI occurs, which is dependent on the nerve growth factor and its receptors, the neurotrophic tropomyosin receptor kinase A and p75 neurotrophin receptor.^[^ ^11^ ^]^ Wong et al. demonstrated that SCs can directly contact pancreatic cancer cells, inducing tumor cells to form projections and migrate toward nerves. This process relies on the expression of the neural cell adhesion molecule by SCs. Additionally, SCs can disperse tumor cells by inserting into them, promoting tumor metastasis.^[^ ^12^ ^]^ SCs activated by tumor cells can also form channels, enhancing the motility of tumor cells and facilitating tumor invasion.^[^ ^13^ ^]^

The colon and rectum are innervated by intrinsic enteric neurons, extrinsic efferent nerves, and afferent nerves. The enteric nervous system (ENS) consists of myenteric and submucosal ganglia, containing various types of enteric neurons and glial cells. Axons arising from the ENS and from extrinsic neurons provide innervation to regulate intestinal functions.^[^ ^14^ ^]^ Although the specific mechanisms of interaction between nerves and colorectal cancer (CRC) cells have not been fully elucidated, PNI is considered an important prognostic factor in CRC. In most studies, the incidence of PNI in CRC patients ranges from 9% to 33% and is associated with an increased risk of local recurrence, distant metastasis, and unfavorable prognosis.^[^ ^15^, ^16^ ^]^ PNI is also correlated with aggressive phenotypes of CRC, including larger tumor size, deeper tumor infiltration, lymph node involvement, poor differentiation, and the presence of distant metastasis. Studies show varying rates of PNI in different stages of CRC: ≈10% in stage I‐II, up to 30% in stage III, and as high as 40% in stage IV. ^[^ ^17^ ^]^ Overall, PNI is a significant factor in CRC progression and represents a major challenge for effective cancer treatment.

The melanoma‐associated antigen (MAGE) A family is a group of genes that encode proteins called cancer‐testis antigens (CTAs).^[^ ^18^ ^]^ These proteins are normally expressed in the testis and placenta but are also expressed in various cancers.^[^ ^18^ ^]^ Immunohistochemical studies have demonstrated MAGEA expression in early‐developing central nervous system (CNS) and PNS, suggesting a potential role in neural system development.^[^ ^19^ ^]^ As biomarkers, MAGEA family proteins are expressed in various malignant tumors, including melanoma, head and neck squamous cell carcinoma, lung cancer, breast cancer, ovarian cancer, liver cancer, and CRC.^[^ ^20^, ^21^, ^22^, ^23^, ^24^, ^25^, ^26^ ^]^ Their expression is associated with poor prognosis, advanced clinical staging, and adverse treatment outcomes. Melanoma‐associated antigen A6 (MAGEA6) is a member of the MAGEA family and has been found to be involved in promoting cancer cell survival and proliferation by inhibiting autophagy in tumor cells.^[^ ^27^, ^28^ ^]^ In gliomas and renal cell carcinomas, knocking down *MAGEA6* expression leads to increased AMP‐activated protein kinase (AMPK) phosphorylation and inhibition of the mTOR pathway, suppressing tumor cell growth.^[^ ^29^, ^30^ ^]^ MAGEA6 can also enhance the activity of E3 ubiquitin ligases, activate downstream signaling pathways, and ultimately lead to ubiquitination and degradation of AMPK.^[^ ^31^ ^]^ However, the biological functions and regulatory mechanisms of MAGEA6 in CRC remain unclear, and its role in PNI has not been reported.

Our investigation reveals novel mechanistic insights into MAGEA6‐mediated PNI in CRC. Knockdown of *MAGEA6* expression significantly inhibited the PNI capability of CRC cells both in vitro and in vivo. MAGEA6 interacts with the transcription factor Yin‐Yang 1 (YY1), which subsequently increases the secretion of CXCL1 by CRC cells, facilitates the recruitment of CRC cells to SCs, while also enhancing tumor cell invasiveness through epithelial‐mesenchymal transition (EMT), ultimately promoting PNI.

## 2 Results

### 2.1 PNI Impacts the Prognosis of CRC Patients

**Figure 1:**
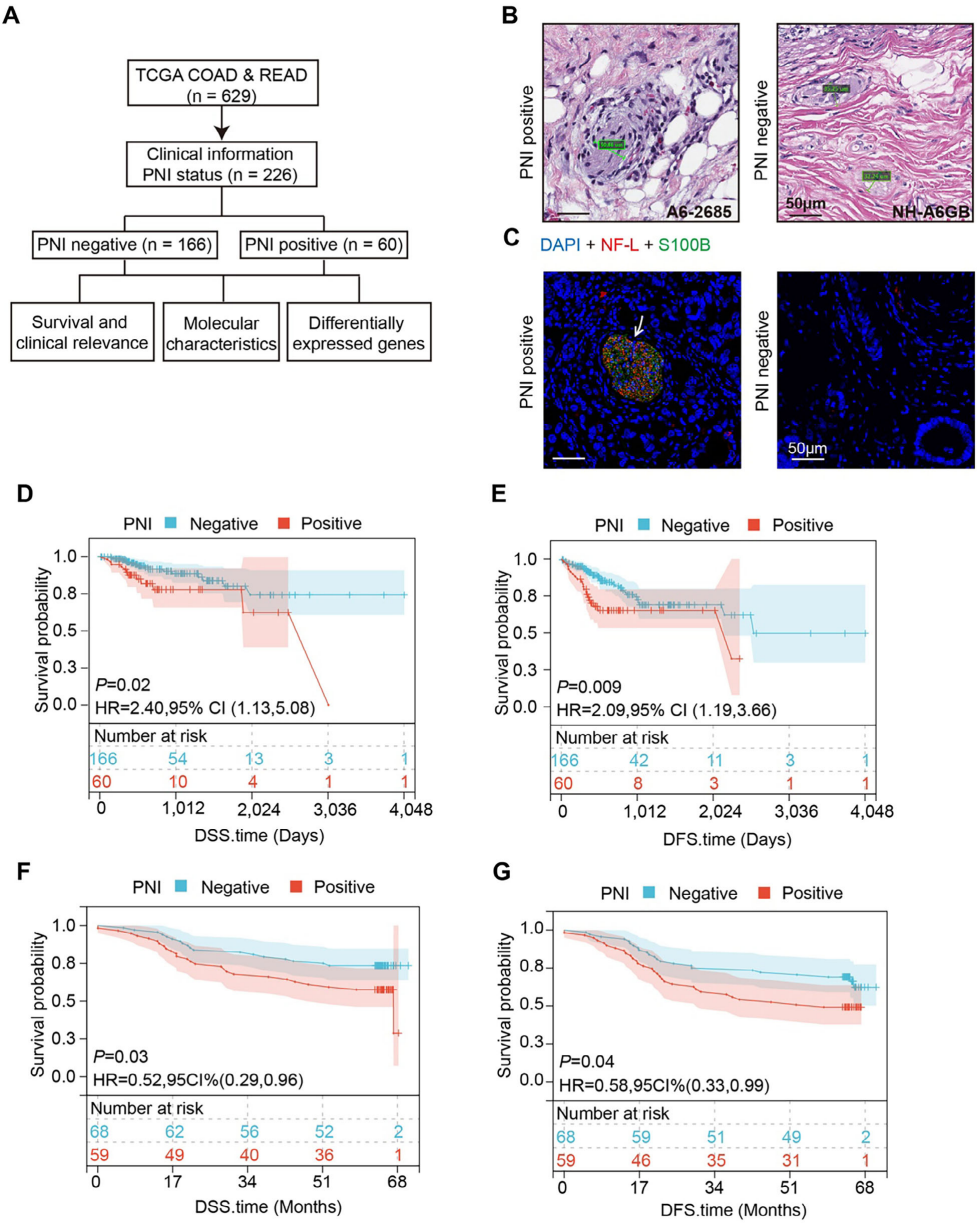
Impact of PNI on CRC patient survival. A) Flowchart of data collection and analysis from TCGA. B) Representative H&E images of PNI‐positive and PNI‐negative tissues; scale bar, 50 µm. C) Representative immunofluorescence images of PNI‐positive and PNI‐negative tissues: NF‐L (red) labels neurons, S100B (green) labels Schwann cells; the arrow indicates tumor cells invading the nerve sheath; scale bar, 50 µm. D, E) Kaplan‐Meier survival analysis assesses the impact of PNI on patient DSS and DFS in the TCGA database. F, G) Kaplan‐Meier survival analysis assesses the impact of PNI on patient DSS and DFS in the Ren Ji cohort.

To assess the impact of PNI on the prognosis of CRC patients, clinical data and Hematoxylin and Eosin staining (H&E) images from the Cancer Genome Atlas (TCGA) database were analyzed, including 173 cases of colon adenocarcinoma (46 PNI‐positive) and 53 cases of rectal adenocarcinoma (14 PNI‐positive) (**Figure** **1**A,B). The occurrence of PNI showed no statistically significant differences among tumors from different locations (Figure S1A,B, Supporting Information). Survival analysis comparing PNI‐negative versus PNI‐positive patients revealed that PNI notably impacted both Disease‐Specific Survival (DSS) and Disease‐Free Survival (DFS) in CRC patients (Figure 1D,E). Correlation analysis indicated that PNI status was significantly associated with tumor invasion depth, distant metastasis, and the 8th edition of the American Joint Committee on Cancer (AJCC) stage (Figure S1C–E and Table S3, Supporting Information). However, no significant correlation was observed between PNI and lymph node metastasis (Figure S1F, Supporting Information).

To validate these findings, a cohort of 127 CRC samples from Ren Ji Hospital was analyzed for PNI using immunofluorescence (IF) staining to detect neurofilament light chain (NF‐L) and the myelin sheath marker S100B to identify nerves and SCs. This revealed 59 cases of PNI (Figure 1C). Survival analysis confirmed that the presence of PNI significantly affected both DSS and DFS in this cohort (Figure 1F,G). Furthermore, larger tumor volume was associated with a higher likelihood of PNI occurrence (Figure S1G, Table S4, Supporting Information). These results indicate that PNI‐positive predicts a poorer prognosis in CRC patients.

### 2.2 Neurotropic Molecular Signature of PNI in CRC

Pathway enrichment analysis in the TCGA cohort was performed to further understand the molecular features of PNI. Kyoto Encyclopedia of Genes and Genomes (KEGG) annotation indicated that the most significantly enriched pathway in PNI‐positive patients was the “Axon guidance pathway”, confirming the effectiveness of the grouping. Gene Set Enrichment Analysis (GSEA) also showed enrichment of tumor‐related pathways such as EMT in PNI‐positive patients (Figure S2A,B, Supporting Information). Additionally, the ESTIMATE algorithm was utilized to calculate the stromal and immune scores, reflecting the stromal and immune cell components in the TME. The stromal score was significantly higher in PNI‐positive patients, whereas immune scores showed no significant difference between the two groups (Figure S2C, Supporting Information). Further analysis of immune cell and stromal cell infiltrates revealed increased infiltration of macrophages, cancer‐associated fibroblasts, and endothelial cells in the TME of PNI‐positive patients (Figure S2D, Supporting Information). These findings suggest that PNI may be associated with stromal cells and macrophages in the TME.

**Figure 2:**
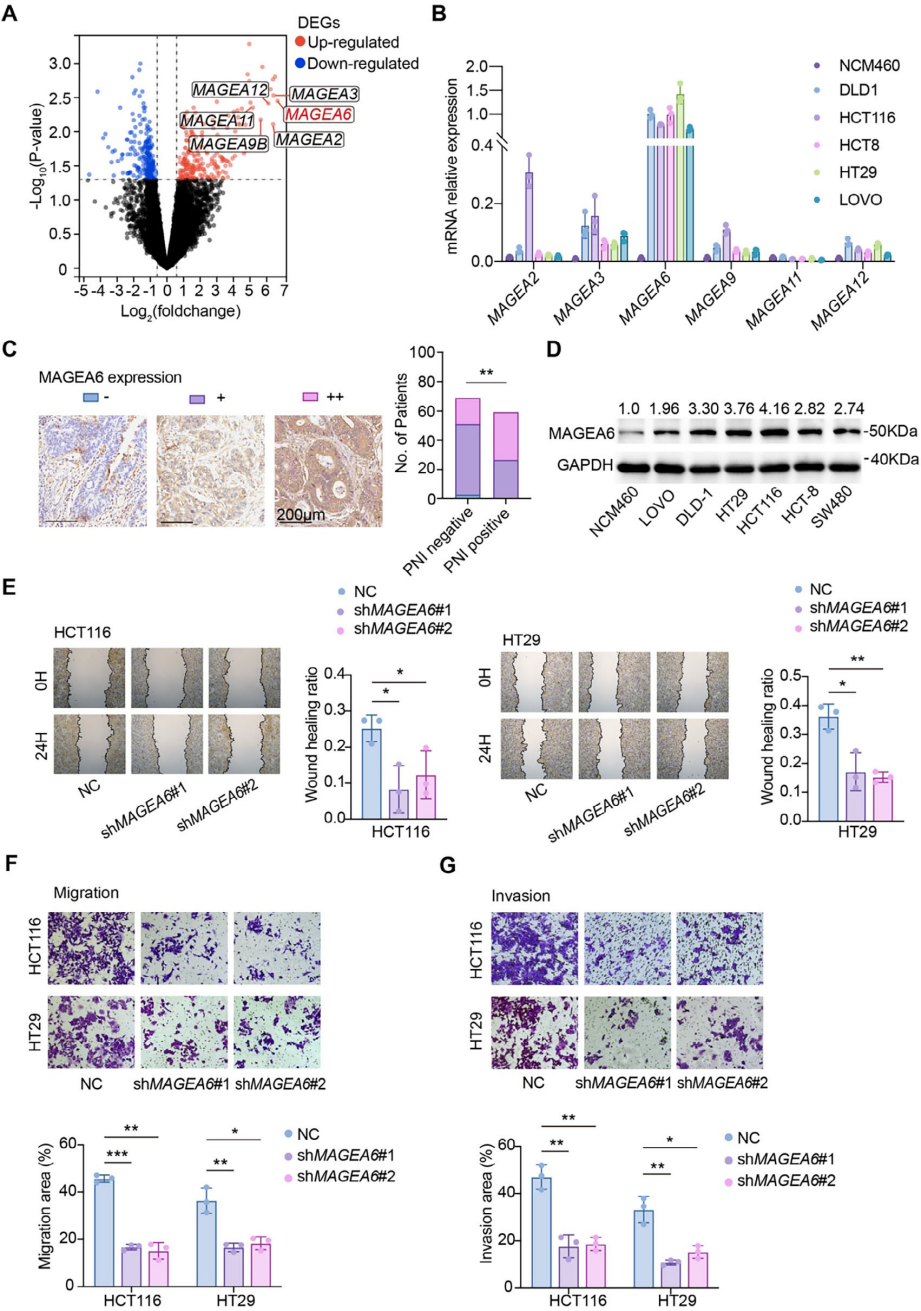
Screening of PNI‐related genes and functional validation in CRC cells. A) The volcano plot shows PNI‐related differentially expressed genes (DEGs); red dots indicate up‐regulated DEGs, while blue dots represent down‐regulated DEGs. B) qPCR validation of *MAGEA* gene mRNA expression levels in normal colon epithelial cells (NCM460) and CRC cell lines. C) IHC analysis of MAGEA6 expression levels in PNI‐positive and PNI‐negative groups, with correlation analysis; scale bar, 200 µm. D) WB analysis of MAGEA6 protein expression in NCM460 and CRC cell lines, with densitometry values. E) Wound healing assay assessed the migration ability of HCT116 and HT29 cells after *MAGEA6* knockdown. F, G) Transwell assays validated the migration and invasion abilities of HCT116 and HT29 cells after *MAGEA6* knockdown. (*p < 0.05, **p < 0.01, ***p < 0.001).

To explore the molecular mechanisms underlying PNI in CRC, differential gene expression analysis was conducted between the PNI‐positive and PNI‐negative patients from the TCGA database. The results revealed that 202 genes were upregulated and 167 genes were downregulated in PNI‐positive patients (Table S5, Supporting Information). Notably, six of the top ten upregulated genes in PNI‐positive patients belonged to the MAGEA family, with *MAGEA6* showing the most significant upregulation (**Figure** **2**A). To validate these findings, the mRNA levels of MAGEA family genes were examined in NCM460 and various CRC cell lines, confirming that *MAGEA6* exhibited the highest expression (Figure 2B). Immunohistochemistry (IHC) was then performed on cancer tissue microarrays (TMAs) from the Ren Ji cohort (*N* = 127) to assess MAGEA6 expression.

The H‐score was calculated for each patient's IHC result, where an H‐score <100 was considered negative (–), 100 <H‐score <200 was considered positive (+), and an H‐score >200 was considered strong positive (++). Correlation analysis between PNI status and MAGEA6 expression levels showed that higher MAGEA6 expression was associated with PNI, which corroborated the findings from the TCGA database (Figure 2C). Besides, survival analysis in the Ren Ji cohort indicated that high MAGEA6 expression was associated with shortened DSS (Figure S2E, Supporting Information). These results suggest that MAGEA6 may be a critical regulator of PNI and is associated with poor patient outcomes in CRC.

### 2.3 Expression and Oncogenic Role of MAGEA6 in CRC

To investigate the role of MAGEA6 in CRC, protein expression levels of MAGEA6 were evaluated across various CRC cell lines and the normal colon epithelial cell line NCM460. MAGEA6 expression was found to be higher in CRC cell lines compared to NCM460 (Figure 2D). Among these, HCT116 and HT29 cells were selected for establishing stable *MAGEA6* knockeddown lines (sh*MAGEA6*), while NCM460 cells were transfected with *MAGEA6* overexpression plasmids (Figure S3A,B, Supporting Information). Given the gene homology within the MAGEA family, RNA expression levels of other MAGEA genes were examined to exclude complementary impacts of variations in these genes on tumor cell functions (Figure S3C,D, Supporting Information). Subsequently, cell proliferation assays, including CCK‐8 and colony formation assays, demonstrated that MAGEA6 did not significantly affect CRC cell proliferation in vitro (Figure S3E–G, Supporting Information). However, wound healing and Transwell assays revealed a notable decrease in cell migration and invasion capabilities upon *MAGEA6* knockdown (Figure 2E–G). Conversely, overexpression of MAGEA6 in NCM460 cells led to enhanced migration and invasion capabilities (Figure S4A,B, Supporting Information). To validate these findings, stable *Magea6* knockdown cell lines (sh*Magea6*) were established in mouse colon cancer cell line MC38, yielding similar results (Figure S4C–E, Supporting Information). Together, these findings suggest that MAGEA6 plays a critical role in promoting the migration and invasion of CRC cells in vitro.

**Figure 3:**
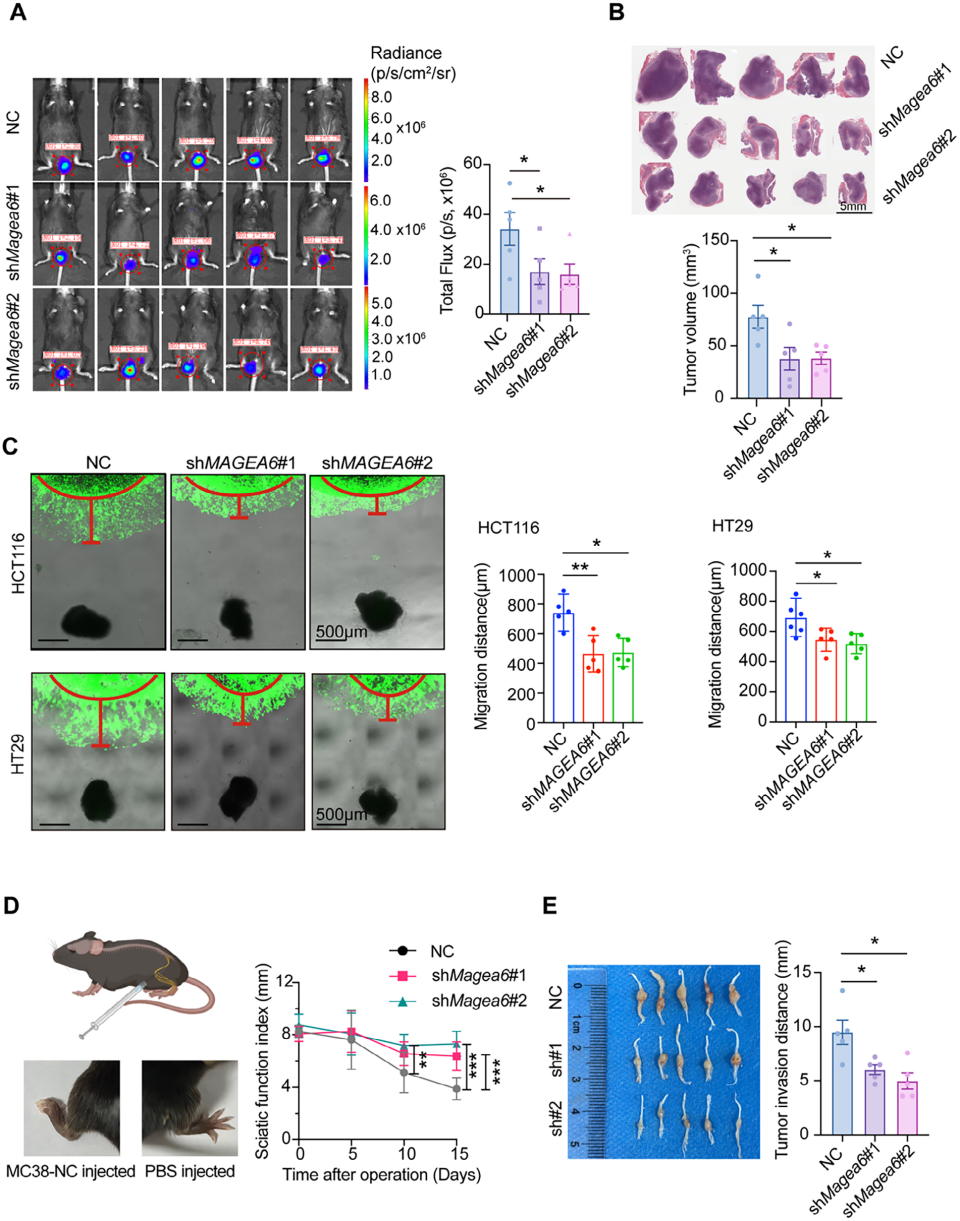
Knockdown of *MAGEA6* expression inhibits CRC growth and PNI. A) In vivo imaging and fluorescence intensity comparison of murine rectal intraepithelial neoplasia models. The radiance (p/s/cm^2^/sr) is shown in the left panel and the statistical chart representing total fluorescence intensity (Total flux, p/s) is shown in the right panel. B) H&E staining of tumor tissues from murine rectal intraepithelial neoplasia models. Tumor volumes were calculated using the formula: V = 1/2 × L × W^2^, where **L** is the longest diameter and **W** is the width perpendicular to **L**. C) Co‐culture of CRC cells with DRG to assess CRC cell neuroinvasive ability. D) Schematic representation of the sciatic nerve model in mice and comparison of sciatic functional index. E) Macroscopic observation and tumor volume comparison of sciatic nerve tumors in mice. (*p < 0.05, **p < 0.01, ***p < 0.001).

Furthermore, a murine rectal orthotopic transplantation tumor model was established by injecting NC or sh*Magea6* MC38 cells into the rectal submucosa of C57BL/6 mice. In vivo imaging confirmed a significant reduction in tumor growth rate in the sh*Magea6* group compared to the control group (**Figure** **3**A). After the 21‐day experimental period, tumor tissues were collected, and H&E staining revealed that the tumor volume in the sh*Magea6* groups was smaller than in the NC group (Figure 3B). In conclusion, both in vitro and in vivo experiments demonstrated the significant impact of MAGEA6 on CRC cells, underscoring its crucial role in tumor progression.

### 2.4 MAGEA6 Facilitates PNI of CRC Cells

To examine the role of MAGEA6 in the PNI of CRC cells, in vitro co‐culture experiments were conducted with CRC cells and dorsal root ganglion (DRG). The sh*MAGEA6* cells exhibited a significant reduction in migration distance toward DRG, indicating impaired PNI capability (Figure 3C; Figure S4F, Supporting Information). In vivo, a sciatic nerve invasion model was established to mimic the process of tumor cell invasion along nerves. Sciatic nerve function was assessed using sciatic functional index, which was significantly higher in the sh*Magea6* group, suggesting better sciatic nerve function compared to the control group (Figure 3D). After sacrificing the mice, the sciatic nerve and tumor were dissected, revealing a significantly shorter distance of neural invasion in the sh*Magea6* group compared to the control (Figure 3E, Figure S4G, Supporting Information).

In the rectal orthotopic transplantation tumor model, IHC staining for the neural marker protein gene product 9.5 (PGP9.5) showed deeper tumor cell invasion and more nerve disruption in the NC group compared to the sh*Magea6* group, which exhibited fewer instances of PNI (Figure S4H, Supporting Information). These results provide further evidence that *MAGEA6* knockdown attenuates CRC cell invasion along nerves.

### 2.5 MAGEA6 Enhances the Recruitment of SCs by CRC Cells

In the DRG, neurons are tightly enveloped by immunologically active SCs. During the co‐culture of DRG and CRC cells, SCs migration from the DRG toward tumor cells and the establishment of contact between them were observed. This interaction potentially facilitates the migration of tumor cells toward the DRG (Figure S5A, Supporting Information). To further investigate the relationship between SCs and CRC in the TME, single‐sample Gene Set Enrichment Analysis (ssGSEA) was used to calculate the infiltration scores of SCs in CRC patients from the TCGA database. Survival analysis revealed that increased infiltration of immature SCs was associated with shorter survival in patients with PNI, while a higher proportion of differentiated SCs correlated with longer survival (Figure S5B,C, Supporting Information). When patients were categorized into high and low MAGEA6 expression groups, the high MAGEA6 group exhibited significantly increased infiltration of immature SCs and decreased infiltration of differentiated SCs (Figure S5D, Supporting Information). These findings suggest a potential association between MAGEA6 expression and SC infiltration within the tumor neural microenvironment.

**Figure 4:**
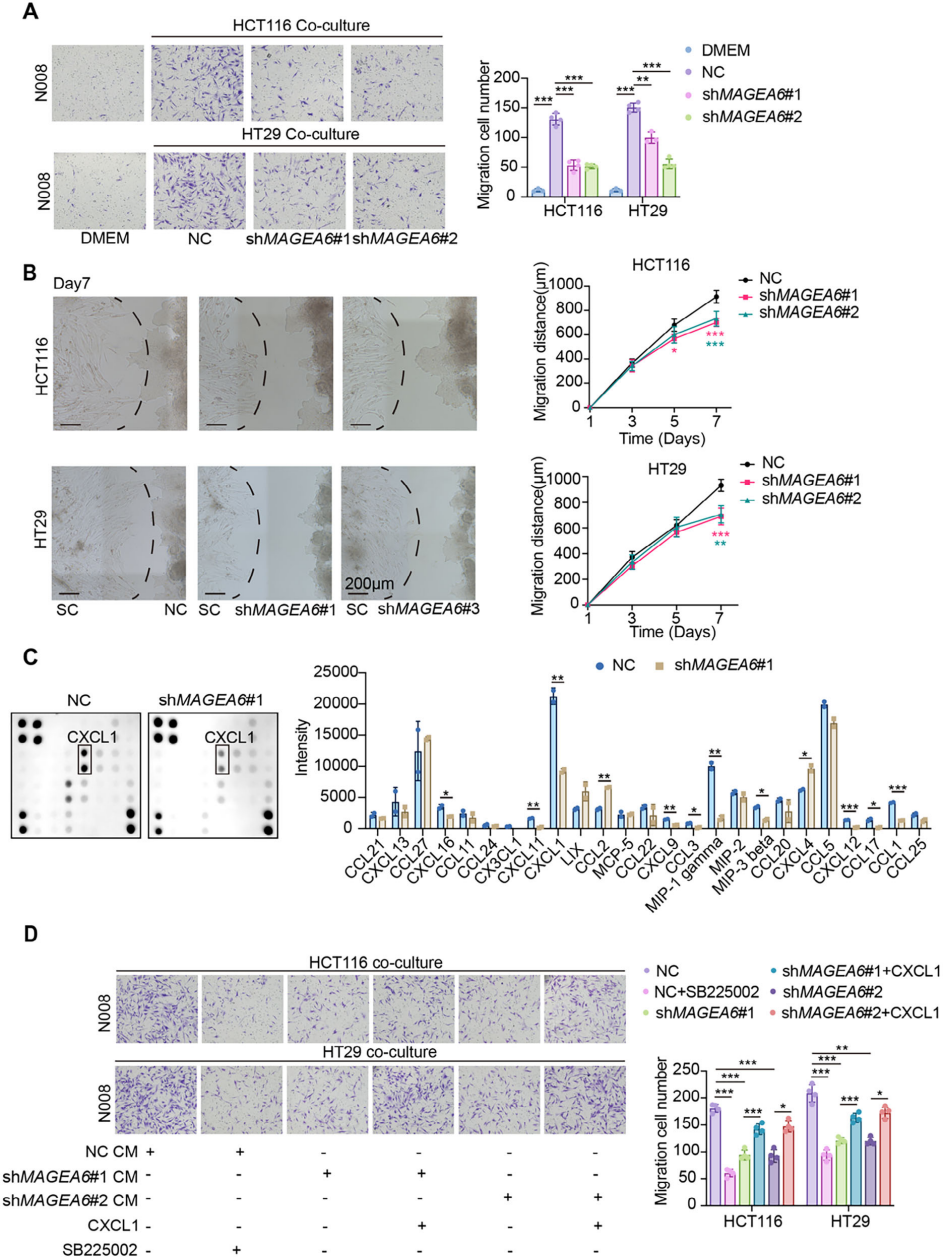
MAGEA6 enhances SCs recruitment by CRC cells. A) Transwell chamber co‐culture of CRC cells with SCs to compare the migration of SCs. B) Matrigel co‐culture of CRC cells with SCs to compare the maximal distance of SCs migration toward CRC cells. C) Chemokine array analysis and quantitative assessment of HCT116 CM. D) Transwell assay with recombinant CXCL1 protein (10 ng mL^−1^) and CXCR2 inhibitor (SB225002, 10 nM) to validate the recruitment role of CXCL1/CXCR2 on SCs. (*p < 0.05, **p < 0.01, ***p < 0.001).

To investigate the impact of MAGEA6 on SCs, SCs were treated with conditioned medium (CM) from NC or sh*MAGEA6* CRC cells. While the proliferative capacity of SCs remained unchanged, their migratory ability decreased when treated with CM from sh*MAGEA6* cells compared to CM from NC cells (Figure S5E,F, Supporting Information). Additionally, Transwell and Matrigel co‐culture experiments between SCs and CRC cells revealed that NC cells had a strong recruiting effect on SCs, whereas *MAGEA6* knockdown significantly attenuated this effect (**Figure** **4**A,B), indicating that MAGEA6 expression in CRC cells influences the recruitment of SCs.

Chemokine array analysis of CM from NC and sh*MAGEA6* CRC cells showed a significant reduction in the release of several chemokines, including CXCL16, CXCL11, CXCL1, CXCL9, CCL3, MIP‐1, MIP‐3, CXCL12, CCL17, and CCL1 following *MAGEA6* knockdown, while CCL2 and CXCL4 levels increased (Figure 4C). Of these, CXCL1, which was highly expressed in the NC group, showed a marked decrease after *MAGEA6* knockdown, a finding further confirmed by ELISA and qPCR (Figure S6A,B, Supporting Information). IF staining confirmed the expression of CXCL1 receptor, CXCR2, on SCs (Figure S6C, Supporting Information). In the co‐culture system of SCs with sh*MAGEA6* CRC cells, the addition of recombinant human CXCL1 protein successfully rescued the recruitment of SCs. Conversely, in the co‐culture system of SCs with NC CRC cells, the addition of the CXCR2 inhibitor SB225002 (10 nM) significantly inhibited SC recruitment without affecting the viability of SCs or CRC cells (Figure 4D; Figure S6D, Supporting Information). These results provide compelling evidence that MAGEA6 enhances the recruitment of SCs by CRC cells through the CXCL1‐CXCR2 axis.

### 2.6 MAGEA6 Interaction with YY1 Regulates CXCL1 Transcription

As shown in Figure S6B (Supporting Information), qPCR demonstrated a decrease in *CXCL1* mRNA levels in sh*MAGEA6* CRC cells. To further investigate how MAGEA6 regulates *CXCL1* transcription, we utilized publicly available ChIP‐Seq databases (Signaling Pathway Project and ChIP‐Atlas) to identify transcription factors that potentially bind to the *CXCL1* promoter region in human digestive system tissues. The Signaling Pathway Project predicted 56 transcription factors, while the ChIP‐Atlas predicted 367 transcription factors (Table S6, Supporting Information).

To explore proteins interacting with MAGEA6, Co‐IP assays were performed. SDS‐PAGE gel electrophoresis and mass spectrometry analysis revealed 467 proteins interacting with MAGEA6 in HCT116 cells and 525 proteins in HT29 cells (Table S6, Supporting Information). A Venn diagram illustrated the intersection of proteins interacting with MAGEA6 with transcription factors predicted to bind to the *CXCL1* promoter, identifying two candidates: YY1 and TCF7L2 (Figure S6E, Supporting Information). Knockdown experiments showed that silencing *YY1* led to a significant decrease in both *CXCL1* mRNA and secreted protein levels in CRC cells, whereas silencing *TCF7L2* had no significant effect (Figure S6F–H, Supporting Information).

**Figure 5:**
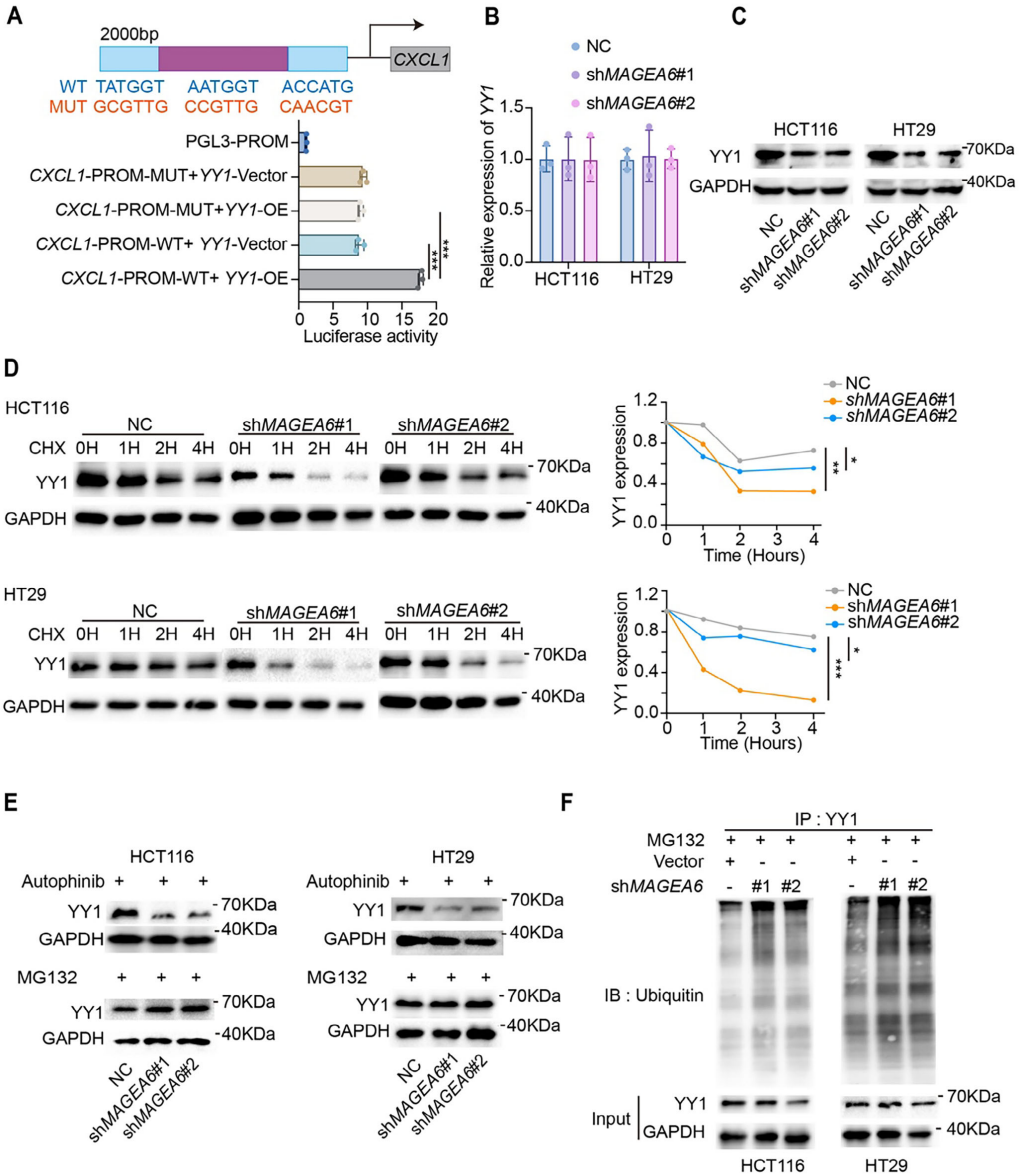
MAGEA6 inhibits YY1 ubiquitination and promotes CXCL1 transcription. A) Luciferase reporter assay to detect YY1 binding to the *CXCL1* promoter region. B, C) qPCR and WB measured mRNA and protein expression levels of YY1 in sh*MAGEA6* and NC CRC cells. D) WB detected YY1 protein degradation levels. E) WB detected YY1 protein levels in sh*MAGEA6* and NC CRC cells treated with autophinib or MG132. F) Co‐IP and WB measured ubiquitination levels of YY1 protein in sh*MAGEA6* and NC CRC cells. (*p < 0.05, **p < 0.01, ***p < 0.001).

To further confirm the involvement of YY1 in regulating *CXCL1* transcription, a luciferase reporter assay was performed. The wild type (WT)‐*CXCL1*‐promoter luciferase reporter plasmid and mutant (MUT)‐*CXCL1*‐promoter luciferase reporter plasmid (containing three mutant sites) were transfected into HCT116 cells overexpressing *YY1* or vector plasmids. The results showed that in YY1‐overexpression cells, WT‐*CXCL1*‐promoter activity was significantly higher than that of the MUT‐*CXCL1*‐promoter (**Figure** **5**A). These findings suggest that MAGEA6 interacts with YY1 to regulate *CXCL1* transcription, thus influencing both mRNA and protein levels of CXCL1 in CRC cells.

### 2.7 MAGEA6 Inhibits YY1 Ubiquitination and Degradation

To investigate the mechanism by which MAGEA6 regulates YY1, we first validated the RNA and protein expression levels of YY1 in sh*MAGEA6* and NC CRC cells. While no significant difference was observed at the mRNA level, YY1 protein levels were significantly lower in sh*MAGEA6* cells (Figure 5B,C). Next, we assessed YY1 protein degradation dynamics by treating cells with cycloheximide (CHX, 20 µg mL^−1^) and collecting samples at 0, 1, 2, and 4 h. WB analysis revealed that YY1 protein degradation was accelerated in the sh*MAGEA6* cells compared to NC cells (Figure 5D). To determine the degradation pathway, cells were treated with the autophagy inhibitor autophinib (1 µM) or the proteasome inhibitor MG132 (10 µM). MG132 treatment restored YY1 protein levels in sh*MAGEA6* cells, while autophinib had no such effect (Figure 5E). Additionally, Co‐IP experiments revealed increased ubiquitination of YY1 in sh*MAGEA6* cells compared to NC cells (Figure 5F), suggesting that MAGEA6 regulates YY1 protein stability through the ubiquitin‐proteasome degradation pathway.

Ubiquitination involves the attachment of ubiquitin to target proteins, which is catalyzed by E1 activating enzymes, followed by E2 transfer and E3 ligase‐mediated attachment. The ubiquitinated proteins are then recognized by the proteasome and degraded. This process can be reversed by deubiquitinases, such as ubiquitin‐specific proteases (USPs), which remove ubiquitin to prevent degradation or alter protein localization.^[^ ^34^, ^35^ ^]^ Mass spectrometry analysis identified a potential interaction between MAGEA6 and USP10. WB validation showed no significant difference in USP10 expression between sh*MAGEA6* and NC cells (Figure S6I, Supporting Information). Although USP10 expression remained unchanged between sh*MAGEA6* and NC cells (Figure S6I, Supporting Information), *USP10* knockdown resulted in decreased YY1 protein levels (Figure S6J, Supporting Information). Co‐IP experiments showed reduced interaction between YY1 and USP10 in sh*MAGEA6* cells compared to NC cells (Figure S6K,L, Supporting Information). These findings suggest that MAGEA6 promotes USP10‐mediated deubiquitination of YY1, thereby stabilizing YY1 protein levels.

### 2.8 YY1 Promotes EMT in CRC Cells

**Figure 6:**
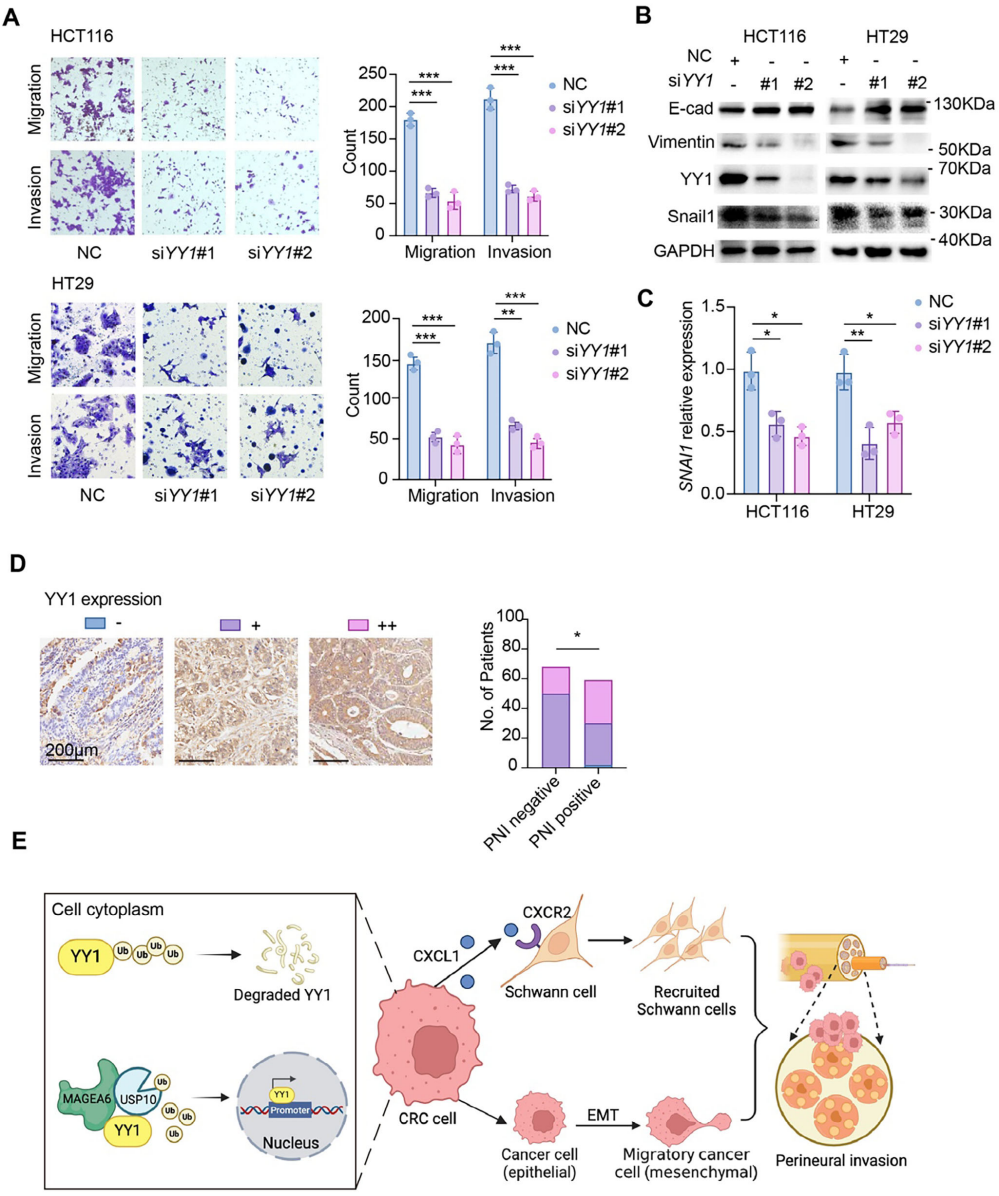
YY1 promotes EMT in CRC cells. A) Transwell assay detected migration and invasion abilities of HCT116 and HT29 cells after *YY1* knockdown. B) WB analysis of EMT‐related proteins in HCT116 and HT29 cells after *YY1* knockdown. C) qPCR detected *SNAI1* mRNA levels in HCT116 and HT29 cells after *YY1* knockdown. D) IHC assessment of YY1 expression levels in human CRC TMA and correlation analysis with PNI. E) Proposed mechanism model of MAGEA6 promoting PNI in CRC through YY1/CXCL1‐SCs recruitment axis and enhancing CRC cell invasion ability through EMT. (*p < 0.05, **p < 0.01, ***p < 0.001).

Given MAGEA6's role in tumor migration and invasion, we investigated whether YY1 contributes to these processes. Transwell assays showed that *YY1* knockdown significantly reduced CRC cell migration and invasion (**Figure** **6**A). Further analysis revealed that *YY1* knockdown increased the expression of E‐cadherin (an epithelial marker) and decreased that of Vimentin (a mesenchymal marker) (Figure 6B), indicating that YY1 promotes EMT in CRC cells, potentially through Snail1 (Figure 6B,C), a key regulator of EMT. IHC staining of YY1 in CRC TMAs from the Ren Ji cohort showed that high YY1 expression correlated with PNI, lymphatic metastasis, and distant metastasis (Figure 6D; Table S7, Supporting Information), indicating its association with poor prognosis in CRC patients.

## 3 Discussion

PNI in CRC is indicative of aggressive tumor behavior and poor prognosis.^[^ ^36^ ^]^ Understanding the cellular and molecular mechanisms that regulate PNI in CRC is crucial for developing targeted therapies. In this study, we demonstrated that: 1) CRC patients with PNI showed significantly shorter DSS and DFS, and PNI correlated with tumor infiltration depth, distant metastasis, AJCC stage, and tumor size; 2) MAGEA6 was identified as a critical regulator of PNI in CRC. Knockdown of *MAGEA6* significantly reduced CRC cell migration, invasion, and PNI both in vitro and in vivo; 3) MAGEA6 promotes YY1 deubiquitination, enhancing CXCL1 expression and SC recruitment, while also driving tumor cell EMT (Figure 6E). To the best of our knowledge, this is the first report demonstrating that MAGEA6 facilitates PNI in CRC.

PNI is now routinely included in pathological reports for CRC. The 8th edition of the AJCC staging guidelines recognizes PNI as a prognostic factor in CRC, with PNI positivity correlating with poorer patient outcomes.^[^ ^37^ ^]^ Additionally, the National Comprehensive Cancer Network Clinical Practice Guidelines classify PNI as a high‐risk factor for CRC recurrence and recommend postoperative chemotherapy for stage II patients with PNI.^[^ ^38^, ^39^ ^]^ Adjuvant chemotherapy has been shown to improve survival outcomes for PNI‐positive patients, extending the 5‐year DFS for stage II‐III CRC patients.^[^ ^40^, ^41^, ^42^ ^]^ Lymph node metastasis is another critical pathological feature associated with poor prognosis and increased cancer‐related mortality in CRC. Some studies suggest a link between PNI and lymph node metastasis, with better 5‐year survival rates observed in CRC cases without perineural or lymphatic infiltration.^[^ ^43^, ^44^, ^45^ ^]^ This may be particularly relevant given that large‐caliber axons frequently interact with lymph nodes. However, the mechanisms of PNI and lymph node metastasis have been predominantly studied independently, and further research is necessary to explore their potential correlation.

Our study revealed that in CRC patients with PNI, several MAGEA family members exhibit upregulated expression, with MAGEA6 showing the highest and most significant increase. MAGEA6, a member of the CTA family, is typically expressed only in the testes and placenta, where it plays essential roles in spermatogenesis and embryonic development.^[^ ^46^ ^]^ However, its expression in tumors has been increasingly recognized as a potential cancer biomarker. For instance, MAGEA6 is highly transcribed and expressed in esophageal cancer, serving as a novel biomarker for diagnosis and treatment.^[^ ^47^ ^]^ In gastric cancer, elevated *MAGEA6* mRNA levels correlate positively with matrix metalloproteinase 9 (MMP‐9) expression, and higher MAGEA6 expression is associated with worse patient prognosis. Furthermore, MAGEA6 protein levels in primary lesions predict the likelihood of recurrence.^[^ ^48^ ^]^ These findings support the hypothesis that MAGEA6 may also act as a biomarker and an indicator of malignant phenotype in CRC. In our study, we observed that sh*MAGEA6* CRC cells exhibit reduced migration and invasion capabilities in vitro, with no significant changes in proliferation. Concurrently, in vivo experiments confirmed slower tumor cell growth in the sh*MAGEA6* group. These findings are not contradictory but likely stem from the complex TME in vivo, where immune cells, stromal cells, and neurons collectively influence tumor progression. In hepatocellular carcinoma, MAGEA6 has been implicated in predicting TME status, immune genomic expression, and response to chemotherapeutic agents.^[^ ^49^ ^]^ In primary non‐small cell lung cancer, patients with cachexia show upregulation of the MAGEA6, MMPs, and EMT pathways.^[^ ^50^ ^]^ Mechanistically, MAGEA6‐derived epitopes tend to elicit a T helper type 2 (Th2)‐type response in tumor patients, whereas healthy individuals typically exhibit a mixed or strongly polarized T helper type 1 (Th1) response to MAGEA6 peptides.^[^ ^51^ ^]^ Th1‐type CD4^+^ T cell help is critical for the induction and maintenance of antitumor cytotoxic T lymphocyte responses. In contrast, Th2‐CD4^+^ T cells may subvert Th1 responses, providing a microenvironment conducive to tumor progression.^[^ ^52^, ^53^ ^]^ Additionally, MAGEA6 has been implicated in modulating protein ubiquitination by enhancing the activity of E3 ubiquitin ligases, leading to the ubiquitination and degradation of AMPK.^[^ ^31^ ^]^ Our study builds on this by demonstrating that MAGEA6 interacts with the deubiquitinase USP10, inhibiting the ubiquitination and degradation of YY1 protein, further emphasizing its significant role in ubiquitination regulation.

Through ssGSEA analysis of CRC samples from the TCGA database, we assessed the enrichment scores of SCs, immature SCs, and differentiated SCs. SCs originate from neural crest‐derived Schwann cell precursors, which initially differentiate into immature SCs and further differentiate into two functionally distinct types: myelinating and non‐myelinating SCs.^[^ ^54^ ^]^ Under conditions such as neural injury and inflammation, both myelinating and non‐myelinating SCs can reprogram into repair SCs, secreting neurotrophic and chemotactic factors that guide axon regeneration.^[^ ^55^ ^]^ Interestingly, gene profiling studies have identified tumor gene sets exhibiting SC‐like characteristics, indicating that tumors can activate SCs.^[^ ^56^, ^57^, ^58^ ^]^ Similar to neural injury, tumor‐activated SCs can reprogram into a dedifferentiated state, characterized by upregulation of immature SC markers and downregulation of mature SC markers.^[^ ^56^ ^]^ Tumor‐activated SCs have been shown to express higher levels of glial fibrillary acidic protein (GFAP), and the presence of GFAP^+^ SCs near tumor cells correlates with poorer prognosis in pancreatic cancer.^[^ ^13^, ^59^ ^]^ Our findings similarly revealed that increased infiltration of immature SCs in PNI‐positive CRC patients was associated with shorter survival, whereas a higher proportion of mature SCs correlated with longer survival. These results suggest that tumor‐activated SCs may promote tumor progression and contribute to poor outcomes in CRC patients.

CXCL1 and its receptor CXCR2 are widely expressed in both the CNS and PNS.^[^ ^60^ ^]^ Upon nerve damage, the activated CXCL1/CXCR2 axis recruits neutrophils and macrophages, facilitating inflammatory responses and nerve repair.^[^ ^61^ ^]^ CXCR2 serves as the primary receptor for CXCL1, but it can also be activated by other chemokines such as CXCL2, CXCL3, CXCL5, CXCL6, CXCL7, and CXCL8.^[^ ^62^ ^]^ Therefore, a more effective therapeutic approach targeting the CXCL1/CXCR2 axis is to focus on CXCR2, thereby simultaneously blocking the effects of CXCL1 and other chemokines. The CXCR2 antagonist SB225002 has shown anti‐tumor activity in several cancers. For example, it reduces cell viability in oral squamous cell carcinoma, inhibits proliferation and migration in intrahepatic cholangiocarcinoma, and suppresses the growth of mouse cholangiocarcinoma tumors.^[^ ^63^, ^64^ ^]^ In our study, inhibition of CXCR2 with SB225002 confirmed that CRC cell recruitment of SCs is dependent on the CXCL1/CXCR2 axis. Moreover, CXCL1 supplementation partially rescued SC recruitment in *MAGEA6*‐knockdown cells. Given the parallels between PNI and nerve injury, where SC recruitment is a shared feature, targeting the CXCL1/CXCR2 axis may serve as a promising strategy to inhibit PNI occurrence.

YY1 is aberrantly expressed in various cancers and is closely linked to cancer progression, metastasis, drug resistance, and poor prognosis.^[^ ^65^, ^66^ ^]^ While YY1 plays a pivotal role in tumor progression, its regulatory mechanisms vary across cancer types.^[^ ^67^ ^]^ In CRC, YY1 suppresses the expression of tumor suppressor miR‐500a‐5p through a p300/YY1/HDAC2‐dependent mechanism, thereby promoting cancer progression.^[^ ^68^, ^69^ ^]^ A recent study highlighted that USP7 maintains high YY1 levels by interfering with its K63‐linked ubiquitination, promoting its oncogenic function.^[^ ^70^ ^]^ Our study revealed that YY1 degradation was accelerated in sh*MAGEA6* cells, with increased ubiquitination, implicating the deubiquitinase USP10 in this process. These findings provide a novel explanation for the abnormal expression of YY1 in CRC cells. YY1 also acts as a pleiotropic factor interacting with various factors in the transcription initiation complex of tumor‐related genes, including *SNAI1*.^[^ ^71^, ^72^ ^]^ Snail1, a key transcription factor, suppresses metastasis‐suppressor proteins such as E‐cadherin and Claudins while promoting mesenchymal markers like N‐cadherin, Vimentin, and Fibronectin, thereby inducing EMT and enhancing the migratory and invasive capabilities of tumor cells.^[^ ^73^, ^74^ ^]^ Interventions targeting YY1 expression or activity may disrupt EMT, inhibiting tumor migration and invasion.^[^ ^75^ ^]^ Our study validated that downregulation of YY1 expression led to reduced EMT in CRC cells, which resulted in decreased migration and invasion capabilities in vitro. Furthermore, YY1 expression was associated with lymph node and distant metastasis, underscoring its clinical significance in CRC.

## 4 Conclusion 

In CRC, MAGEA6 upregulates *CXCL1* transcription through interaction with YY1, facilitating SC recruitment and the occurrence of PNI. Concurrently, YY1 promotes EMT, enhancing tumor cell invasion and migration. Targeting the MAGEA6/YY1/CXCL1‐SC axis may provide a promising strategy to inhibit PNI and tumor progression, ultimately improving patient prognosis.

## 5 Experimental Section

### Data Collection and Analysis

Transcriptomic profiles and clinical data of CRC patients were obtained fromTCGA database using the “TCGAbiolinks” R package. Representative H&E histopathological images depicting PNI were downloaded from the TCGA online portal (https://portal.gdc.cancer.gov/).

Kaplan‐Meier survival curve analysis was performed using the “survival” R package, with comparisons conducted using the log‐rank test. Hazard ratios were calculated using the Cox proportional hazards regression model. Data visualization was conducted using the “ggplot2” R package. Immune and stromal scores for TCGA patients were calculated using the “ESTIMATE” R package, representing the infiltration levels of immune and stromal cells in tumor tissues. Infiltration scores for five immune cell types (B cells, CD4^+^ T cells, CD8^+^ T cells, macrophages, and natural killer cells) and two stromal cell types (endothelial cells and cancer‐associated fibroblasts) in the TME were determined using the “EPIC” R package. Pathway enrichment analysis was performed using the KEGG and GSEA. Differential expression analysis for individual genes was conducted using the “DESeq2” R package with the criteria of |log_2_ (fold‐change) | > 1 and *p *< 0.05 for differential expression gene selection.

### Clinical Specimens

This study included 127 patients who underwent surgical resection of primary CRC at Ren Ji Hospital, Shanghai Jiaotong University (Shanghai, China), between April and August 2017. Follow‐up continued until October 2023. None of the patients had received neoadjuvant radiotherapy or chemotherapy prior to surgery. All clinical specimens were collected with approval from the Ethics Review Committee of Ren Ji Hospital (No. KY2021‐120‐B).

### Cell Lines and Cell Culture

Human CRC cell lines HCT116, HT29, DLD1, LOVO, SW480, and HCT8 were obtained from the Cell Bank of the Chinese Academy of Sciences (Shanghai, China). The human normal colonic epithelial cell line NCM460 and murine colon cancer cell line MC38 were obtained from Otwo Biotech (Shenzhen, China), while the human primary Schwann cell line HUM‐iCELL‐N008 was purchased from iCell Saibai (Shanghai, China). Cell line identity was confirmed using STR genotyping. All human CRC cell lines and NCM460 were cultured in McCoy's 5A medium (16600082, Gibco, USA) supplemented with 10% fetal bovine serum (FBS, A5670701, Gibco, USA) and 1% penicillin/streptomycin (15140148, Gibco, USA), and maintained at 37 °C in a humidified incubator with 5% CO_2_. MC38 and N008 cell lines were cultured in Dulbecco's modified Eagle's medium (DMEM, 11965092, Gibco, USA) supplemented with 10% FBS and 1% penicillin/streptomycin. All experiments were performed with mycoplasma‐free cells.

### Lentiviral Transduction

The lentiviral particles used in this study, including the design of interference sequences, transfection, and virus collection, were provided by GenePharma (Shanghai, China). The detailed sequences of the shRNAs were as follows: sh‐*MAGEA6*#1, GCAAAGCTTCCGATTCCTTGC; sh‐*MAGEA6*#2, GGAGGAGCTGAGTGTGTTAGA; sh‐*Magea6*#1, TGCTGTTGACAGTGAGCGACG; sh‐*Magea6*#2, TGCTGAGGACTGAGCGACTCA. CRC cells were seeded into 6‐well plates at appropriate densities. Lentiviral particles were transduced into the CRC cells according to the lentiviral transduction protocol provided by GenePharma, using Polybrene (GenePharma, Shanghai, China) as the transfection reagent. After 24 h of transfection, the medium was replaced with a regular culture medium. After 1–2 passages, puromycin (540222, Sigma, USA) was added to select transduced cells. The cells were maintained with regular changes of the antibiotic‐containing medium to select a population stably transduced with lentiviral particles.

### Small Interfering RNA (siRNA) Transfection

CRC cells were seeded in 6‐well plates to achieve 60% fusion. A total of 10 nM siRNA was mixed with 200 µL of jetPRIME Buffer (101000046, Polyplus, France) and vortexed for 10 s. Next, 4 µL of jetPRIME Reagent (101000046, Polyplus, France) was added, and vortexed again for 10 s. The mixture was then incubated at room temperature for 10 min. The resulting siRNA‐transfection reagent mixture was added dropwise to the cell culture medium, and the cells underwent a media change 6 h post‐transfection. RNA or protein samples were collected 48 h post‐transfection for validation of transfection efficiency. The siRNAs oligonucleotides used in this study were synthesized from GenePharma (Shanghai, China) and the detailed sequences of the siRNAs are listed as follows: si‐*YY1*#1, AAGUCCAGCAACAGGAAGAUC; si‐*YY1*#2, UCAUCGAGGUCAACGGGAUCA; si‐*TCF7L2*#1, GCGUCUCAGCUGGAUCAAAUU; si‐*TCF7L2*#2, UGAAGGUGCUGGAUACAUUUU si‐*USP10*#1, GCCCUGAUGAAUUCAAUCATT; si‐*USP10*#2, CCUGUGGACUUGGAAAUUATT.

### RNA Extraction and Quantitative Polymerase Chain Reaction (qPCR)

Total RNA was extracted using TRIzol reagent (15596018, Thermo Fisher Scientific, USA) according to the manufacturer's instructions. RNA purity and concentration were assessed spectrophotometrically (NanoDrop 2000, Thermo Fisher Scientific, USA). Reverse transcription was performed with 1 µg RNA using the PrimeScript RT Master Mix (RR057B, Takara, Japan) under the following conditions: 37 °C for 15 min, 85 °C for 5 s. Quantitative PCR amplification was conducted using SYBR Green Premix Ex Taq (B21202, Selleck, USA) on a ViiA7 Real‐Time PCR System (Applied Biosystems, USA). Each 20 µL reaction contained 10 µL SYBR Green mix, 0.4 µM forward/reverse primers (sequences in Table S1, Supporting Information), and 2 µL cDNA template. Thermal cycling parameters: 95 °C for 30 s (initial denaturation), followed by 40 cycles of 95 °C for 5 s and 60 °C for 30 s, with melt curve analysis. Relative mRNA expression was calculated using the 2^−ΔΔCt^ method normalized to *GAPDH*.

### Protein Extraction and Western Blotting (WB)

Cells were lysed in RIPA buffer (P0013B, Beyotime, China) supplemented with protease inhibitors (87785, Thermo Fisher Scientific, USA). Protein concentrations were determined via BCA assay (P0009, Beyotime, China). WB was performed following standard protocols, as described previously.^[^ ^32^ ^]^ Briefly, equal amounts of protein were separated by 10% SDS‐PAGE and transferred to nitrocellulose membranes. After blocking with 5% nonfat milk for 2 h at room temperature, membranes were incubated overnight at 4 °C with primary antibodies (Table S2, Supporting Information), followed by HRP‐conjugated secondary antibodies (1:5000 dilution) for 1 h. Protein bands were visualized using an ECL substrate (P0018S, Beyotime, China) on a ChemiDoc Touch Imaging System (Bio‐Rad, USA).

### IHC and IF

Tissue sections were subjected to antigen retrieval using sodium citrate buffer (P0081, Beyotime, China) at 95 °C for 10 min. Endogenous peroxidase activity was blocked using H_2_O_2_ (ST858, Beyotime, China) for 15 min. After blocking with 5% bovine serum albumin (BSA, A500023‐0100, Sangon Biotech, China), sections were incubated overnight with the corresponding primary antibody (Table S2, Supporting Information) at 4 °C. For IHC, sections were then incubated with the species‐specific horseradish peroxidase (HRP)‐linked secondary antibodies (ShareBio, Shanghai, China) for 1 h at room temperature. The antigen was visualized using diaminobenzidine (DAB, 36201ES03, Yeasen, China), followed by nuclear counterstaining with hematoxylin (C007, Beyotime, China). For IF, sections were incubated with species‐specific fluorescent HRP‐linked secondary antibodies (A‐11008, A‐11004, Thermo Fisher Scientific, USA) for 1 h at room temperature in the dark. The cell nuclei were stained with DAPI (C1002, Beyotime, China) for 5 min. Images were captured using a confocal microscope (Leica, Germany). All slides were independently evaluated and scored by two pathologists based on the proportion of positively stained tumor cells and signal intensity.

### Transwell Assay

For migration assays, 5 × 10⁴ cells in serum‐free medium were seeded into upper chambers of 8 µm pore Transwell inserts (353097, Corning, USA). Lower chambers contained 20% FBS as a chemoattractant. After 24 h incubation, non‐migrated cells were removed with cotton swabs. Migrated cells were fixed with 4% paraformaldehyde (PFA, G1101, Servicebio, China), stained with 0.1% crystal violet (G1114, Servicebio), and quantified under a light microscope (5 random fields/insert).

For invasion assays, inserts were pre‐coated with 50 µL Matrigel matrix (354234, BD Biosciences, USA) 1:10 dilution in serum‐free medium, and polymerized at 37 °C for 1 h. Subsequent steps followed the migration protocol.

### Wound Healing Assay

Confluent CRC monolayers in 6‐well plates were mechanically scratched using a sterile 200 µL pipette tip. The scratched area was washed with a culture medium to remove detached cells, and a fresh culture medium was then added. Wound closure was monitored at 0/24 h using phase‐contrast microscopy (Nikon Eclipse Ti2, Japan). Migration rates were quantified as percentage wound area reduction using ImageJ (v1.8.0) with the MRI Wound Healing Tool plugin.

### Cell Viability Assay

Cells (3 × 10^3^/well) in 96‐well plates were incubated with CCK‐8 reagent (10% v/v, CK04, Dojindo, Japan) for 2 h at 37 °C. Optical density (OD450 nm) was measured using a Synergy H1 microplate reader (BioTek, USA). Proliferation indices were normalized to time‐matched controls.

### Plate Colony Formation Assay

Low‐density seeding (600 cells/well in 6‐well plates) was performed to assess reproductive viability. After a 14‐day culture, colonies were fixed with 4% PFA (G1101, Servicebio, China) and stained with 0.1% crystal violet (G1114, Servicebio, China). Clusters >50 cells were enumerated microscopically (Nikon TS2, 4× objective) with automated counting via ImageJ (v1.8.0).

### In Vitro DRG Co‐Culture Model

The in vitro DRG co‐culture model was established as previously described.^[^ ^33^ ^]^ Balb/c‐n or C57BL/6J mice (4‐6 weeks, male) were anesthetized with 2% isoflurane and sacrificed by CO_2_ asphyxia. After sterilization, a longitudinal incision was made in the center of the mouse's back, separating the skin and subcutaneous fibrous tissue to expose the spine. The thoracic spine was dissected to harvest DRGs. Two incisions along the ventral midline were made to remove the spinal cord. DRGs were isolated from the intervertebral foramina, characterized by their rounded and hyaline appearance, under a stereo microscope. The surrounding sheath and vessels were carefully excised using ophthalmic scissors. The isolated DRGs were placed into a culture dish for further use.

CRC cells were counted and resuspended in Matrigel (356231, Corning, USA). A 5 µL drop of this cell‐gel mixture, containing 5 × 10^4^ cells, was placed 1 mm away from the DRG. The dish was incubated at 37 °C for 2 min to allow Matrigel polymerization. A second 1 µL drop of cell‐free Matrigel was added to bridge the DRG and cancer cells, excluding nonspecific cellular interactions. After another 2 min, the DRG and CRC cells in Matrigel were submersed in 1.5 mL culture medium supplemented with 10% FBS. The PNI capacity of tumor cells was evaluated by measuring the distance of tumor cell migration toward the DRG, with images captured using confocal microscopy (Leica, Germany).

### SCs and CRC Cells Co‐Culture

Transwell chamber co‐culture: CRC cells (5 × 10⁴ cells/well) were seeded in a 24‐well plate and allowed to adhere. SCs (HUM‐iCELL‐N008, 5 × 10⁴) in serum‐free medium were seeded into the upper chamber of 8 µm Transwell inserts (353097, Corning, USA). Migrated cells after 12 h were fixed/stained as per the Transwell assay protocol.

Matrigel co‐culture: 3 × 10^5^ SCs were resuspended in 5 µL of Matrigel (356231, Corning, USA) and added to a well of a 24‐well plate. Meanwhile, 5 µL of Matrigel containing 3 × 10^5^ CRC cells was added 1 mm away from the SCs. The plates were incubated at 37 °C for 2 min to allow Matrigel polymerization. Subsequently, 1 µL of Matrigel was added to bridge the CRC cells and SC cells. After incubating for another 2 min, the CRC and SC cells in Matrigel were submerged in 1.5 mL of culture medium supplemented with 10% FBS. The distance of SCs migration toward CRC cells was observed and photographed using an inverted microscope (Nikon, Japan) to assess the recruitment effect of CRC cells on SCs.

### Enzyme‐Linked Immunosorbent Assay (ELISA)

Conditioned media from serum‐starved (2% FBS, 48 h) CRC cultures were centrifuged (1500 rpm, 5 min), and the supernatant was used immediately for analysis. ELISA was performed according to the manufacturer's instructions (PCDBH0116, P&C, Shanghai, China). Absorbance was measured at 450 nm using a Varioskan LUX multimode microplate reader (Thermo Fisher Scientific, USA). A standard curve was constructed to calculate chemokine levels, and the results were expressed in pg/mL.

### Luciferase Reporter Assay

Luciferase reporter assays were conducted using the Dual‐Glo Luciferase Assay System (GM‐040502A, Genomeditech, Shanghai, China) according to the manufacturer's protocol. Briefly, mutant (GCGTTG, CCGTTG, CAACGT) or wild‐type (TATGGT, AATGGT, ACCATG) *CXCL1*‐promoter luciferase reporter plasmids were constructed by inserting the corresponding *CXCL1* binding motif into the pGL3‐basic vector. The mutant or wild‐type *CXCL1*‐promoter luciferase reporter plasmid was transfected into HCT116 cells that overexpressed YY1 or empty plasmids. A Renilla luciferase plasmid was co‐transfected simultaneously as an internal control. Firefly and Renilla luciferase activities were measured 48 h after transfection using a Multiskan FC automatic fluorescent enzyme labeling instrument (Thermo Fisher Scientific, USA). Each experiment was performed in triplicate.

### Co‐Immunoprecipitation (Co‐IP) Assay

Magnetic Protein A/G Beads (B23202, Bimake, Shanghai, China) were pre‐cleaned using PBS and then incubated with the specified primary antibody or IgG for 20 min in a binding buffer at 25 °C. After three washes with cold lysis buffer, 500 µg pre‐cleared cell lysates (15 000 rpm, 20 min, 4 °C) were incubated with antibody‐conjugated beads overnight at 4 °C. The beads were then washed 3 times with cold lysis buffer and resuspended in 50 µL of 1× SDS‐PAGE loading buffer (P0015A, Beyotime, China). The samples were boiled at 95 °C for 5 min. After centrifugation at 4 °C, 1000 rpm for 5 min to remove the beads, the supernatants were collected for immunoblot detection using the indicated antibodies.

### Rectal Orthotopic Transplantation Tumor Model

All animal experiments in this study complied with the ARRIVE guidelines and were conducted in accordance with the U.K. Animals Act, 1986, and associated guidelines, and were approved by the Ethics Committee of Ren Ji Hospital, affiliated with Shanghai Jiao Tong University (No. RA‐2021‐387). Animals were housed under standard laboratory conditions with a 12 h light‐dark cycle and had access to food and water ad libitum. Animals were monitored for signs of distress or adverse effects throughout the experiment. The study endpoints were predetermined to minimize suffering, and animals were humanely euthanized using CO_2_ inhalation when reaching experimental endpoints.

C57BL/6 mice (6–8 weeks, male) were fasted for 24 h prior to the experiment. After anesthetizing the mice with isoflurane, they were positioned supine, and the rectum was exteriorized using tweezers. Tumor cells (25 µL, 5 × 10⁵ cells per mouse) were injected into the submucosal layer of the rectum using a 1 mL syringe. Following injection, gentle pressure was applied to the injection site to prevent cell leakage, and the rectum was carefully repositioned. After the procedure, the mice were placed on a 37 °C heating pad to recover. After 21 days, the mice were sacrificed, and tumor tissues were collected for further study.

### Sciatic Nerve Invasion Model

Sciatic nerve injections were performed following established protocols.^[^ ^33^ ^]^ Briefly, C57BL/6 mice (6–8 weeks, male) were anesthetized using isoflurane (5% for induction, 1%–3% for maintenance). The sciatic nerves on both sides were exposed, and 5 µL of a cell suspension containing 1 × 10⁵ cells per microliter was injected into the sciatic nerve using a 10 µL microsyringe. The wounds were then closed with surgical sutures. To assess the sciatic nerve function index, the extension length between the first and fifth toes of the hind limbs was measured weekly. Limb function was graded based on the response of the hind limb paw to manual extension of the body, with scores ranging from 5 (normal) to 1 (total paw paralysis). Mice were euthanized 4 weeks postsurgery. Upon euthanasia, the sciatic nerves with tumors were exposed to evaluate the severity of PNI. After 15 days of modeling, the mice were sacrificed and the tumor tissues were collected for further analysis.

### Statistical Analysis

All continuous variables were expressed as mean ± SEM or median. Group comparisons used: Student's t‐test (two‐group parametric), Mann‐Whitney U test (two‐group nonparametric), and Log‐rank test (survival analysis). All statistical analyses were performed using SPSS 26.0 and GraphPad Prism 8.4.0. Two‐sided tests were used for all comparisons, and *p *< 0.05 was considered statistically significant (*p < 0.05, **p < 0.01, ***p < 0.001).

### Declaration of Generative AI and AI‐Assisted Technologies in the Writing Process

During the preparation of this work, the authors used ChatGPT to check for grammatical errors and make appropriate language refinements. After using this tool, the authors reviewed and edited the content as needed and took full responsibility for the content of the publication.

### Informed Consent Statement

Informed consent was obtained from all subjects involved in the study.

## Conflict of Interest

The authors declare no conflict of interest.

## Author Contributions

H.W., K.H., and R.H. contributed equally to this work. H.W. performed an investigation and wrote the original draft. K.H. and R.H. performed an investigation. W.L. and H.W. performed software and visualization. S.Z. and L.‐J.J. performed data curation. J.X., S.‐H.J., and M.Y. performed project administration, and wrote, reviewed, and edited the original draft.

## Supporting information



Supporting Information

## Data Availability

Data sharing is not applicable to this article as no new data were created or analyzed in this study.
