# Supplementary material for: MAGEA6 Engages a YY1‐Dependent Transcription to Dictate Perineural Invasion in Colorectal Cancer
Source: Adv Sci (Weinh). 2025 Mar 27;12(25):2501119. doi: 10.1002/advs.202501119 (PMC12224987; doi:10.1002/advs.202501119)
Supplement: Supplementary file 1 — Supporting Information [file ADVS-12-2501119-s001.docx]

**Supplemental Data**

**MAGEA6 engages a YY1-dependent transcription to dictate perineural invasion in colorectal cancer**

Hao Wang^1#^, Kexin He^1#^, Ruixue Huo^1#^, Weihan Li^1^, Shan Zhang^3^, Lu-Ju Jiang^3^, Hao Wu^1^, Minhao Yu^2^*, Shu-Heng Jiang^3^*, Junli Xue^1^*

^1^Department of Oncology, Shanghai East Hospital, School of Medicine, Tongji University, Shanghai 200092, P.R. China

^2^Department of Gastrointestinal Surgery, Ren Ji Hospital, School of Medicine, Shanghai Jiao Tong University, Shanghai 200127, P.R. China

^3^State Key Laboratory of Systems Medicine for Cancer, Shanghai Cancer Institute, Ren Ji Hospital, School of Medicine, Shanghai Jiao Tong University, Shanghai 200240, P.R. China

^#^Hao Wang, Kexin He, Ruixue Huo contributed equally to this work.

*Corresponding authors: E-mails: 1310666xuejunli@tongji.edu.cn (J. Xue), shjiang@shsci.org (S. Jiang), and yuminhao@renji.com (M. Yu)

**
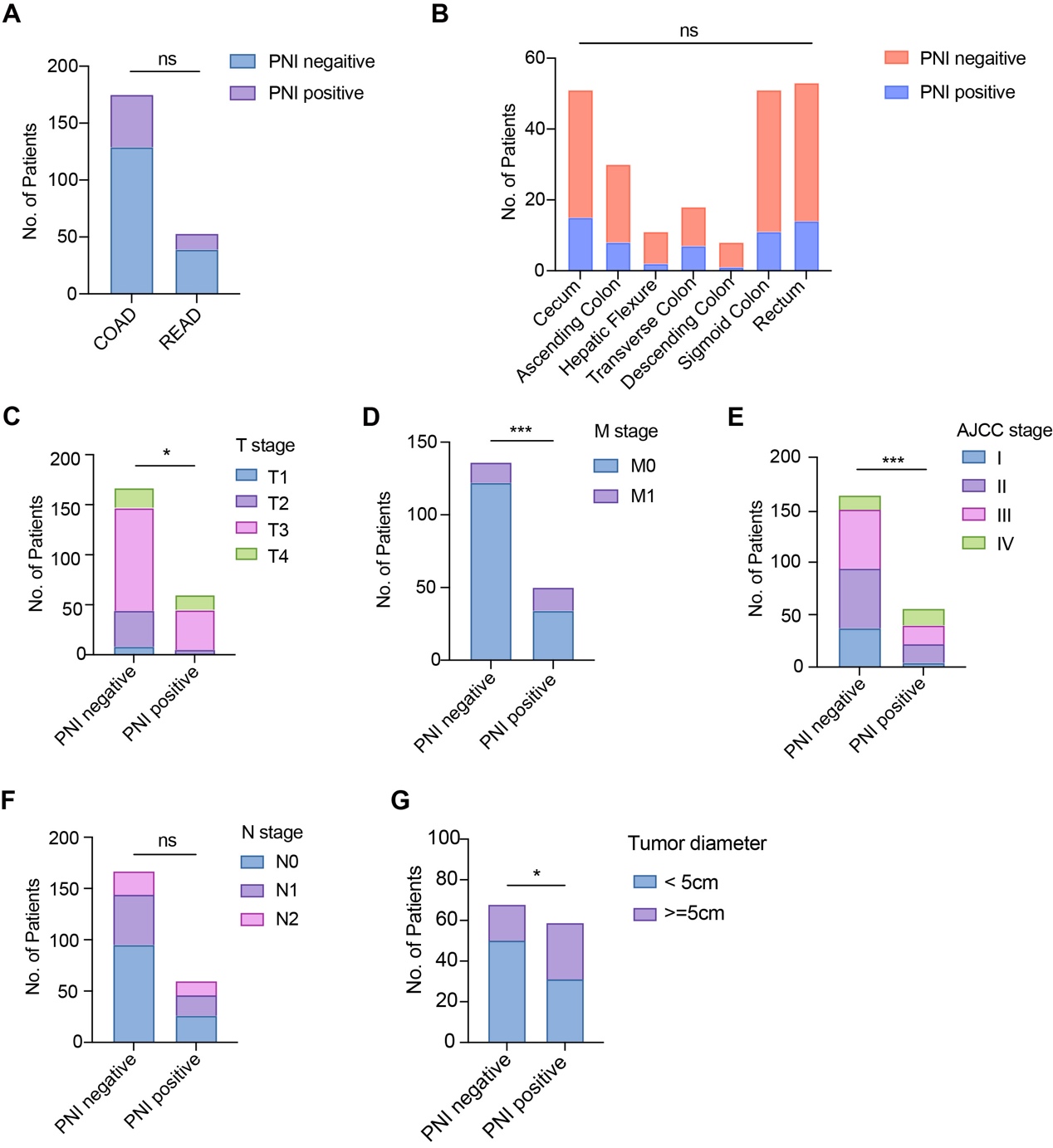
S****upplemental Figures**

Figure S1

(A-B) Comparison of PNI incidence in different tumor locations. (C-G) Comparisons of T stage (C), M stage (D), AJCC stage (E), N stage (F), and tumor diameter (G) in patients grouped by PNI status. (* *p* < 0.05, ** *p* < 0.01, *** *p* < 0.001).


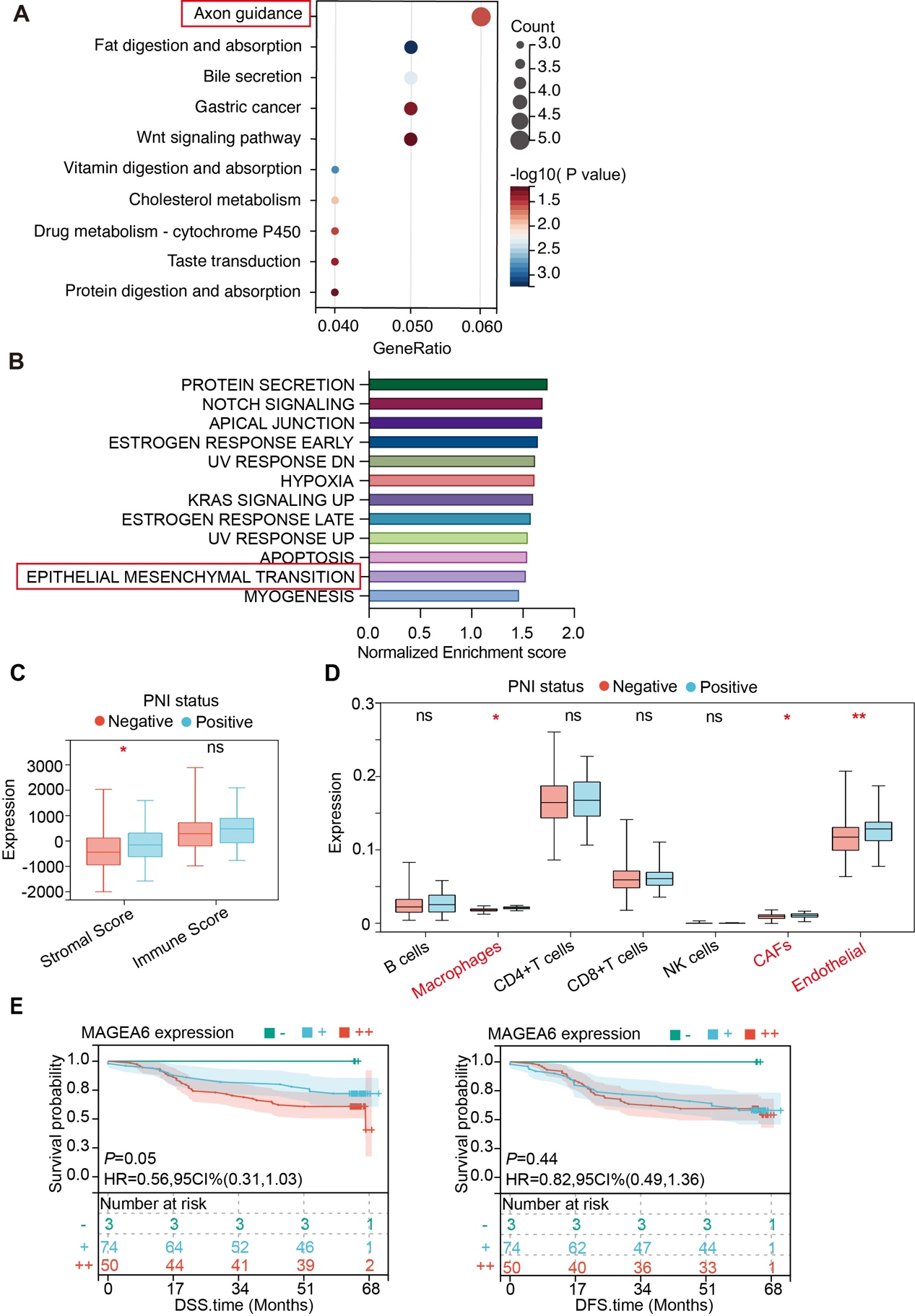


Figure S2

(A) KEGG pathway enrichment analysis in PNI-positive patients. (B) GSEA analysis of upregulated Hallmark signaling pathways in PNI-positive patients. (C) Comparison of stromal and immune scores in CRC patients grouped by PNI status. (D) Analysis of tumor microenvironment cell components grouped by PNI status. (E) Kaplan-Meier survival analysis assessed the impact of MAGEA6 expression on patient DSS and DFS in the Ren Ji cohort. (* *p* < 0.05, ** *p* < 0.01, *** *p* < 0.001).


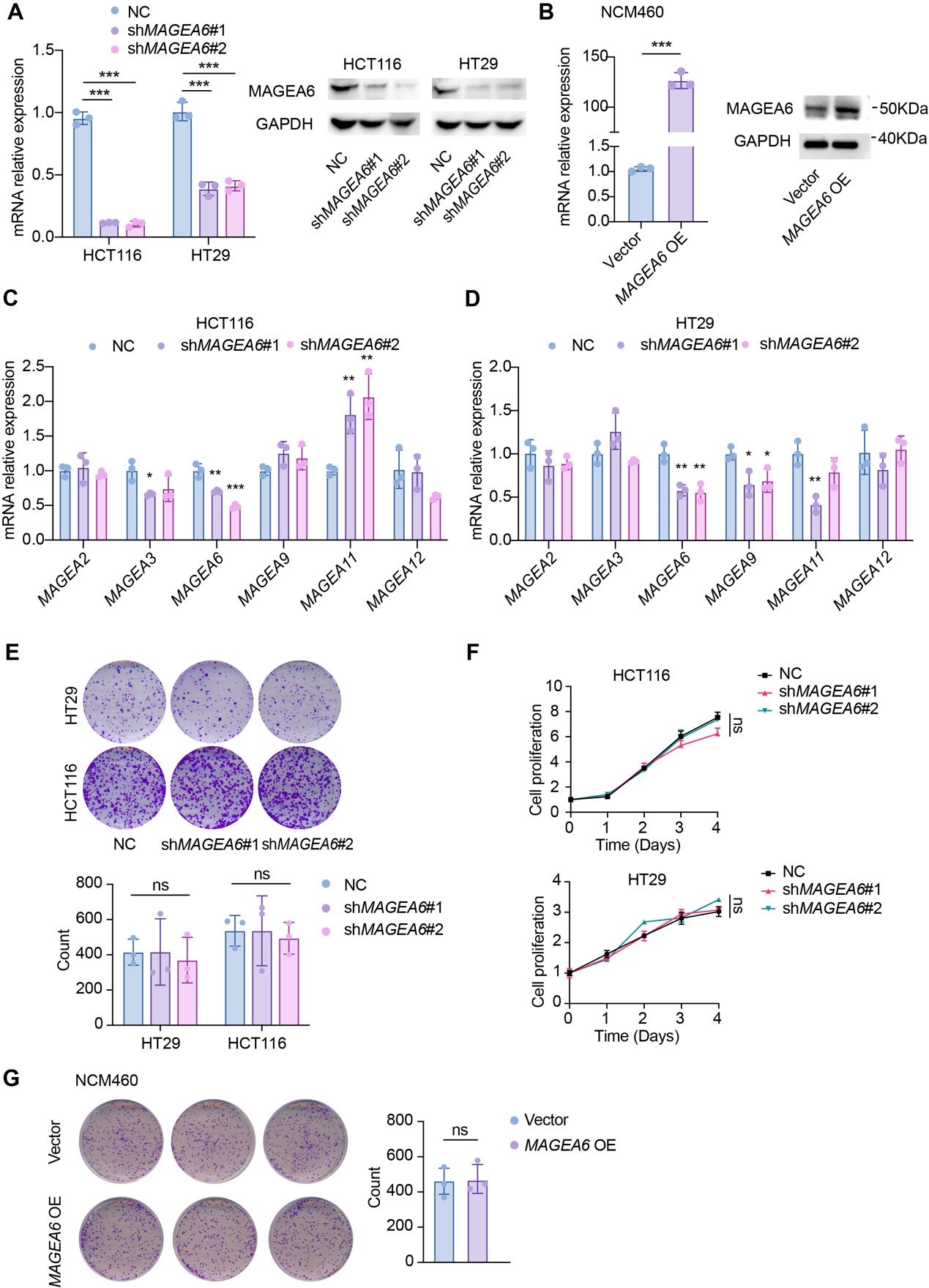


Figure S3

(A) qPCR and WB detected MAGEA6 expression levels in HCT116 and HT29, verifying the transfection efficiency of sh*MAGEA6*. (B) qPCR and WB detected MAGEA6 expression levels in NCM460, verifying the transfection efficiency of *MAGEA6* OE plasmid. (C-D) qPCR validated the mRNA levels of other MAGEA genes in HCT116 and HT29 cells after transfection with sh*MAGEA6*. (E-G) Colony formation assay and CCK8 assay detected proliferation ability of CRC cells and NCM460 cells. (* *p* < 0.05, ** *p* < 0.01, *** *p* < 0.001).


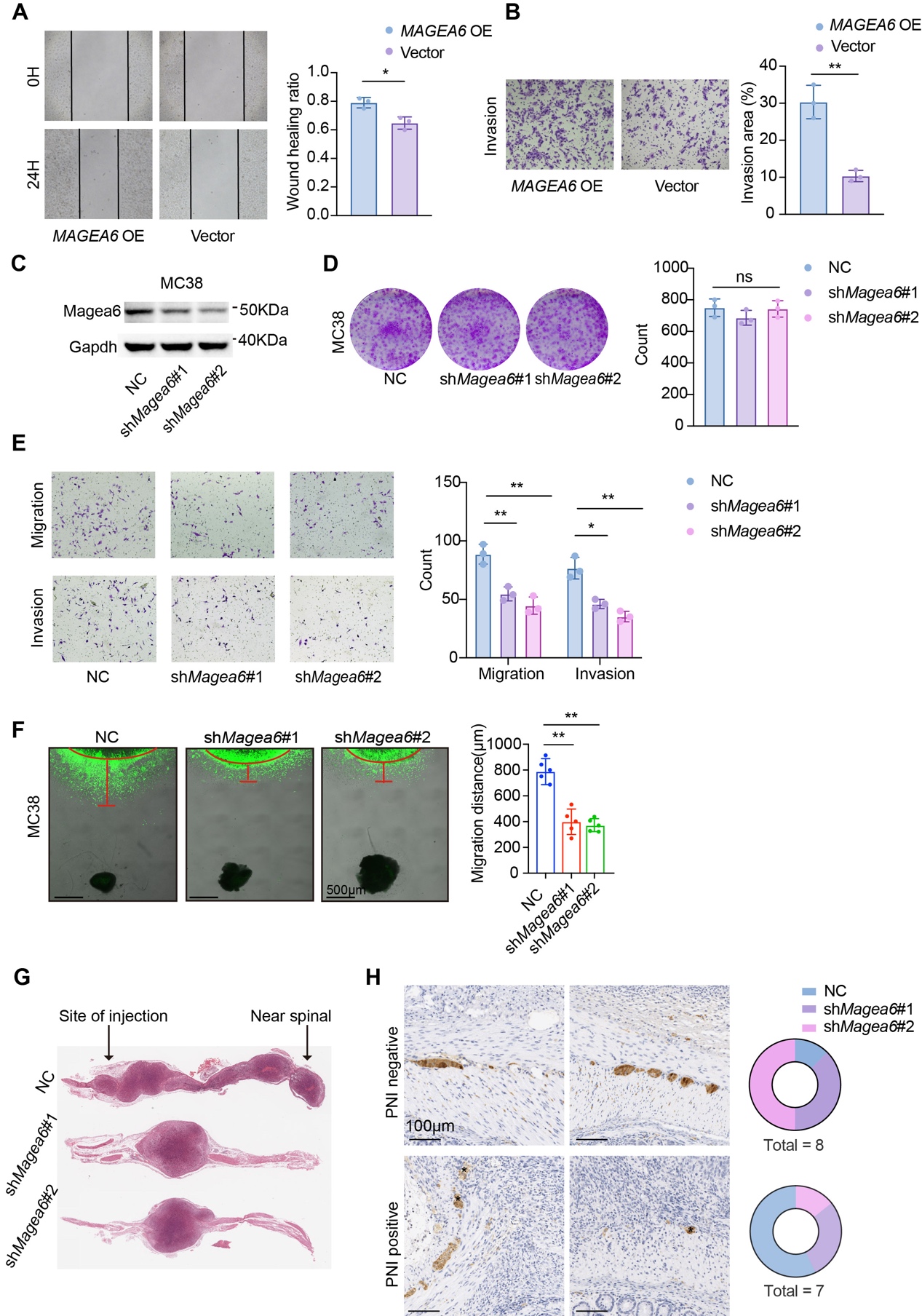


Figure S4

(A) Wound healing assay assessed the migration ability of NCM460 cells after *MAGEA6* over expression. (B) Transwell assays validated the invasion abilities of NCM460 cells after *MAGEA6* over expression. (C) WB validated sh*Magea6* transfection efficiency in MC38 cells. (D) Colony formation assay detected the proliferation ability of MC38 cells. (E) Transwell assay detected the migration and invasion abilities of MC38 cells. (F) Co-culture experiment of murine DRG with CRC cells to evaluate the migration distance of tumor cells towards the DRG. (G) H&E staining showed the invasion distance of the tumor along the sciatic nerve. (H) IHC staining of the nerve marker PGP9.5 to evaluate the occurrence of PNI in murine rectal intraepithelial neoplasia. (* *p* < 0.05, ** *p* < 0.01, *** *p* < 0.001).


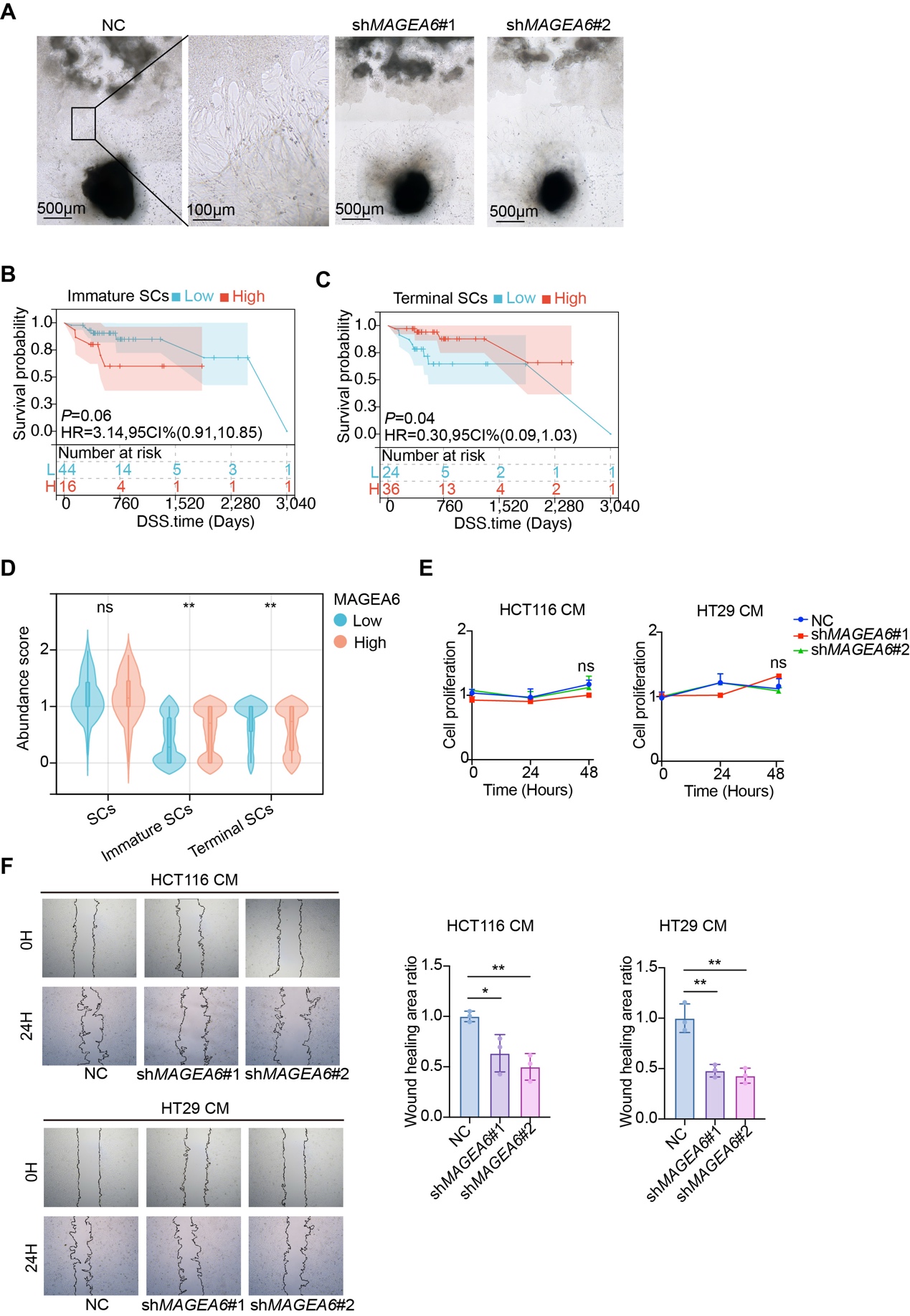


Figure S5

(A) During DRG and tumor cell co-culture experiment, SCs migration from DRG towards tumor cells was observed. (B-C) Kaplan-Meier survival analysis assessing the impact of immature SCs and terminal SCs on patient DSS in the TCGA database. (D) Correlation analysis of MAGEA6 expression levels with SC enrichment scores in the TCGA database. (E) CCK8 assay detected the proliferation ability of SCs after treatment with CRC cell conditioned medium (CM). (F) Wound healing assay evaluated the migration ability of SCs after treatment with CRC cell CM. (* *p* < 0.05, ** *p* < 0.01, *** *p* < 0.001).


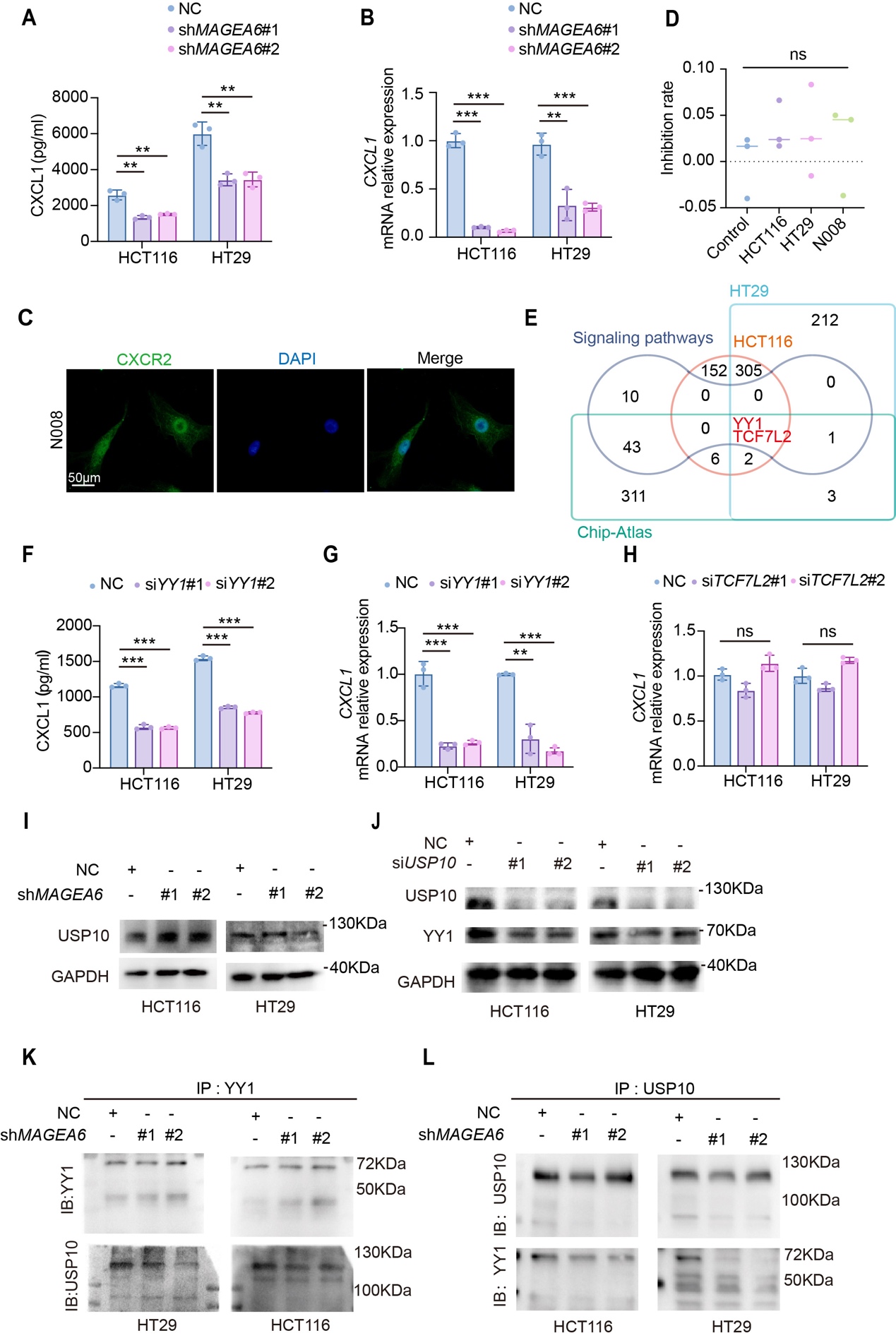
Figure S6

(A-B) ELISA and qPCR detected *CXCL1* mRNA and protein expression levels after *MAGEA6* knockdown. (C) IF validation of CXCR2 expression in SCs. (D) CCK8 assay measured the cell viability of CRC cells and N008 cells after treatment with SB225002 (10 nM) for 24 hours. Inhibition rate = (Control well – Experimental well) / (Control well – Blank well). (E) Venn diagram showed the intersection of MAGEA6 interacting proteins with CXCL1 transcription factors. (F-G) ELISA and qPCR detected CXCL1 mRNA and protein expression levels after YY1 knockdown. (H) qPCR detected *CXCL1* mRNA levels after *TCF7L2* knockdown. (I) WB validated USP10 protein expression levels in sh*MAGEA6* and NC cells. (J) WB detected YY1 protein expression changes after *USP10* knockdown. (K-L) Co-IP validated the interaction between YY1 and USP10 in sh*MAGEA6* and NC cells. (* *p* < 0.05, ** *p* < 0.01, *** *p* < 0.001).

**Supplemental Tables**

Table S1. Primer used in this study

| **Genes** | **Forward primer (5’-3’)** | **Reverse primer (5’-3’)** |
| --- | --- | --- |
| *MAGEA6* | AGGGGAGGGAAGACAGTATCT | AAAGCCCACTCATGCAGGAG |
| *MAGEA2* | \| ATTGCTGCTTGGAACTCGGA \| \| --- \| | CCTCACACACGCTCCTTCTC |
| *MAGEA3* | CGGTGAGGAGGCAAGGTTC | GACTCTGGGGAGGATCTGGT |
| *MAGEA9* | \| CAGGCCTTGGTCTGAGACAG \| \| --- \| | \| TGAGAACCGAACCTTGGAGG \| \| --- \| |
| *MAGEA11* | \| TTGGGCAGGTGAGCACTATG \| \| --- \| | GCCAGGAGGAACCCTCAATC |
| *MAGEA12* | \| GTCAGCCCTGGACAACCTAC \| \| --- \| | CCTACCTCAGGCTTCTCACC |
| *CXCL1* | \| AGCTTGCCTCAATCCTGCAT \| \| --- \| | TTTCTGACCAACGGCTCCAG |
| *YY1* | CCTCTCAGATCCCAAACAACTG | GCCTTTATGAGGGCAAGCTATT |
| *TCF7L2* | ATGAGCCACGAGGAAGGAG | CAGGAGAGAGGAGGAGGAG |
| *SNAI1* | TCGGAAGCCTAACTACAGCGA | AGATGAGCATTGGCAGCGAG |
| *GAPDH* | CTGGGCTACACTGAGCACC | AAGTGGTCGTTGAGGGCAATG |

Table S2. Antibodies used in this study

| **Antibodies** | **Company** | **Dilution** | **Application** |  |
| --- | --- | --- | --- | --- |
| MAGEA6 | Proteintech | 1:2000 | WB |  |
| GAPDH | Proteintech | 1:10000 | WB |  |
| YY1 | Proteintech | 1:5000 | WB |  |
| Ubiquitin | Proteintech | 1:2000 | WB |  |
| USP10 | Proteintech | 1:200 | WB |  |
| E-cadherin | Proteintech | 1:10000 | WB |  |
| Vimentin | Proteintech | 1:2000 | WB |  |
| SNAI1 | Proteintech | 1:1000 | WB |  |
| NF-L | Proteintech | 1:200 | IF |  |
| S100B | Servicebio | 1:500 | IF |  |
| CXCR2 | Proteintech | 1:200 | IF |  |
| YY1 | Proteintech | 1:100 | Co-IP |  |
| MAGEA6 | Santa | 1:50 | Co-IP |  |
| USP10 | Proteintech | 1:100 | Co-IP |  |
| MAGEA6 | | Proteintech | 1:100 | IHC |
| YY1 | Proteintech | 1:1000 | IHC |  |
| Goat anti-Rabbit IgG (H+L) | Proteintech | 1:5000 | WB |  |
| Goat anti-Mouse IgG (H+L) | Proteintech | 1:5000 | WB |  |
| Goat anti-Rabbit IgG (H+L) | Proteintech | 1:200 | IHC |  |
| Goat anti-Mouse IgG (H+L) | Proteintech | 1:200 | IHC |  |
| CY5- Goat anti-Rabbit IgG (H+L) | Servicebio | 1:1000 | IF |  |
| FITC- Goat anti-Mouse IgG (H+L) | Servicebio | 1:1000 | IF |  |

Table S3. Correlation analysis between PNI status and clinical-pathological features of TCGA cohort

| **Characteristics** | **PNI status** | | ***p*** |
| --- | --- | --- | --- |
|  | **Absent (N=166)** | **Present (N=60)** |  |
| **Gender** |  |  | 0.18 |
| Female | 88(38.93%) | 25(11.06%) |  |
| Male | 78(34.51%) | 35(15.49%) |  |
| **Age** |  |  | 0.95 |
| Mean±SD | 63.41±12.99 | 63.55±13.72 |  |
| **BMI** |  |  | 0.24 |
| Mean±SD | 30.43±21.76 | 28.25±6.06 |  |
| **Tumor location** | |  | 1 |
| Colon | 127(56.19%) | 46(20.35%) |  |
| Rectum | 39(17.26%) | 14(6.19%) |  |
| **Grade** |  |  | 0.78 |
| Moderate-High | 145(64.16%) | 54(23.89%) |  |
| Low | 21(9.29%) | 6(2.65%) |  |
| **Vascular invasion** | |  | **6.90e-08** |
| Absent | 140(61.95%) | 29(12.83%) |  |
| Present | 26(11.50%) | 31(13.72%) |  |
| **Tumor invasion depth** | |  | **7.00e-03** |
| T1 | 8(3.54%) | 1(0.44%) |  |
| T2 | 36(15.93%) | 4(1.77%) |  |
| T3 | 103(45.58%) | 40(17.70%) |  |
| T4 | 19(8.41%) | 15(6.64%) |  |
| **Lymph invasion** | |  | 0.1 |
| Absent | 95(42.04%) | 26(11.50%) |  |
| Present | 71(31.42%) | 34(15.04%) |  |
| **Distant metastasis** | |  | **8.30e-04** |
| Absent | 122(53.98%) | 34(15.04%) |  |
| Present | 14(6.19%) | 16(7.08%) |  |
| Unknow | 30(13.27%) | 10(4.42%) |  |
| **AJCC stage** |  |  | **7.30e-04** |
| I | 38(16.81%) | 4(1.77%) |  |
| II | 57(25.22%) | 20(8.85%) |  |
| III | 57(25.22%) | 19(8.41%) |  |
| IV | 14(6.19%) | 17(7.52%) |  |

| Table S4. Correlation analysis between PNI status and clinical-pathological features of Ren Ji cohort | | | |
| --- | --- | --- | --- |
| **Characteristics** | **PNI status** | | ***p*** |
|  | **Absent (N=68)** | **Present (N=59)** |  |
| **Gender** |  |  | 0.27 |
| Female | 29(22.83%) | 19(14.96%) |  |
| Male | 39(30.71%) | 40(31.50%) |  |
| **Age** |  |  | 0.59 |
| <65 | 33(25.98%) | 32(25.20%) |  |
| >=65 | 35(27.56%) | 27(21.26%) |  |
| **Tumor location** |  |  | **0.05** |
| Colon | 29(22.83%) | 36(28.35%) |  |
| Rectum | 39(30.71%) | 23(18.11%) |  |
| **Grade** |  |  | 0.84 |
| Moderate-High | 50(39.37%) | 42(33.07%) |  |
| Low | 18(14.17%) | 17(13.39%) |  |
| **Tumor size (cm)** | |  | **0.04** |
| <2 | 5(3.94%) | 2(1.57%) |  |
| 2~5 | 45(35.43%) | 29(22.83%) |  |
| >=5 | 18(14.17%) | 28(22.05%) |  |
| **T satge** |  |  | 0.55 |
| T1 | 4(3.15%) | 2(1.57%) |  |
| T2 | 12(9.45%) | 6(4.72%) |  |
| T3 | 50(39.37%) | 50(39.37%) |  |
| T4 | 2(1.57%) | 1(0.79%) |  |
| **N stage** |  |  | 0.78 |
| N0 | 40(31.50%) | 32(25.20%) |  |
| N1 | 16(12.60%) | 17(13.39%) |  |
| N2 | 12(9.45%) | 10(7.87%) |  |
| **Distant metastasis** | |  | 1 |
| M0 | 63(49.61%) | 54(42.52%) |  |
| M1 | 5(3.94%) | 5(3.94%) |  |
| **AJCC stage** |  |  | 0.58 |
| I | 13(10.24%) | 6(4.72%) |  |
| II | 27(21.26%) | 25(19.69%) |  |
| III | 23(18.11%) | 23(18.11%) |  |
| IV | 5(3.94%) | 5(3.94%) |  |
| **Tumor recurrence** | |  | 0.07 |
| No | 45(35.43%) | 29(22.83%) |  |
| Yes | 23(18.11%) | 30(23.62%) |  |

| Table S5. Differentially expressed genes related to PNI in CRC patients from TCGA database | | | | |
| --- | --- | --- | --- | --- |
| **Gene** | **Log FC** | **Average Expression** | **t** | ***p*** |
| MAGEA6 | 6.66014063 | -1.7968713 | 3.32781435 | 0.00354947 |
| CSAG2 | 6.50513045 | -3.2657801 | 3.6902986 | 0.0015612 |
| MAGEA3 | 6.41061815 | -1.5441188 | 3.41149507 | 0.00293879 |
| MAGEA2 | 6.39288705 | -1.7628235 | 2.97556639 | 0.00779151 |
| CSAG3 | 6.38469743 | -3.2908455 | 3.64955402 | 0.00171283 |
| GAGE12D | 6.24792653 | -2.7697789 | 3.5037205 | 0.00238518 |
| MAGEA12 | 6.10628786 | -2.3892325 | 3.29261103 | 0.00384215 |
| PTPN20B | 5.74259311 | -2.4303488 | 3.83442438 | 0.00112428 |
| MAGEA9B | 5.6426397 | -3.1727239 | 3.04659082 | 0.00665813 |
| MAGEA11 | 5.17015405 | -3.3679367 | 3.22779804 | 0.0044441 |
| GAGE2A | 5.03038714 | -3.1711759 | 3.3045758 | 0.00374011 |
| GBA3 | 4.95699477 | -2.7463124 | 3.62543606 | 0.00180938 |
| PCSK1N | 4.95322314 | 1.09424727 | 4.17334636 | 0.00051914 |
| TMIGD1 | 4.86069808 | -2.8238444 | 3.45728852 | 0.00264967 |
| NR1H4 | 4.8561038 | -2.7046354 | 3.72595493 | 0.00143949 |
| PAGE1 | 4.72660003 | -3.9528497 | 2.96753888 | 0.00793078 |
| GABRA3 | 4.69748249 | -3.2534765 | 2.56082446 | 0.01914845 |
| NXF2 | 4.62934844 | -2.5096231 | 2.52077209 | 0.0208428 |
| FGB | 4.50804258 | -2.374467 | 3.21041679 | 0.00462064 |
| HSD3B2 | 4.38541127 | -2.5046789 | 2.96921119 | 0.00790157 |
| GAGE4 | 4.32277629 | -3.5884688 | 2.98227843 | 0.00767688 |
| FREM2 | 4.28929268 | -0.8024232 | 3.08223275 | 0.00615133 |
| LGSN | 4.25880245 | -3.6789496 | 2.76399459 | 0.01238116 |
| LRRC24 | 4.24290796 | -1.3886604 | 3.18569333 | 0.00488359 |
| CHGB | 4.10777468 | -2.444837 | 2.86533988 | 0.00992796 |
| APOA4 | 4.09303421 | -3.2672508 | 2.48346672 | 0.02254698 |
| GAGE8 | 4.02065778 | -4.0776414 | 3.16584076 | 0.00510526 |
| ALOX15 | 4.01460475 | -0.083597 | 3.41147983 | 0.00293889 |
| MKRN3 | 3.94758519 | -3.8548556 | 2.441786 | 0.02460531 |
| RSPO4 | 3.81869774 | -2.9492263 | 2.33438242 | 0.0307464 |
| SCNN1B | 3.73780252 | 0.56526204 | 2.65344432 | 0.01571509 |
| GUCA2B | 3.70979516 | -1.8607878 | 2.3886168 | 0.02748633 |
| GUCA2A | 3.69739767 | 1.27213191 | 3.10793014 | 0.0058094 |
| HOXD11 | 3.664906 | -0.9908803 | 2.13484697 | 0.0460635 |
| ZNF280A | 3.63336452 | -3.0361815 | 2.92027439 | 0.00880087 |
| DLX3 | 3.60898135 | -2.5693816 | 2.39894515 | 0.02690316 |
| APOA1 | 3.54525772 | -1.1230378 | 2.20529221 | 0.03999808 |
| CA4 | 3.52617997 | 1.10531202 | 2.41244625 | 0.02615824 |
| SLC5A12 | 3.52072871 | -2.5398317 | 2.32076159 | 0.03161974 |
| DACT2 | 3.408181 | -0.906636 | 2.41728848 | 0.02589579 |
| SP8 | 3.38547087 | -2.668112 | 2.11989315 | 0.04745423 |
| NPY1R | 3.36795744 | -1.809131 | 2.43797453 | 0.02480206 |
| ZNF556 | 3.3653775 | -3.0247888 | 2.22866302 | 0.03815331 |
| HAVCR1 | 3.35598036 | -1.636686 | 2.11836184 | 0.04759877 |
| BEX1 | 3.33708295 | -2.6252892 | 2.43620569 | 0.02489387 |
| DIRAS2 | 3.31824344 | -3.8266085 | 2.39533185 | 0.02710585 |
| HOXD10 | 3.30884413 | -0.3175818 | 2.45651688 | 0.02385853 |
| NBPF22P | 3.24729347 | -2.4098746 | 2.58138509 | 0.01832998 |
| TMEM195 | 3.19728839 | -2.0817773 | 2.47567229 | 0.02291918 |
| SLC17A4 | 3.15752822 | -0.0137089 | 2.98025127 | 0.00771132 |
| MS4A1 | 3.15090109 | -1.5957212 | 2.19779437 | 0.04060703 |
| PRIMA1 | 3.0893326 | -2.4768439 | 2.29693235 | 0.03320306 |
| NEB | 3.08022601 | -1.4531568 | 2.15044934 | 0.04465206 |
| GPC2 | 3.07010357 | -0.5343801 | 2.7158074 | 0.01374176 |
| MEP1B | 3.04845912 | -2.5282769 | 2.66977235 | 0.01517386 |
| FER1L4 | 3.04367475 | 3.20188994 | 2.81219361 | 0.01114963 |
| HOXC9 | 3.03749529 | -2.652706 | 2.21915234 | 0.0388944 |
| BMP3 | 3.03611234 | -2.2521114 | 2.29720692 | 0.0331844 |
| SNORD10 | 3.01399318 | -2.9443456 | 2.5302853 | 0.02042803 |
| REC8 | 3.00281206 | 1.94457221 | 3.31807635 | 0.00362817 |
| IL17A | 2.97782517 | -3.3036804 | 2.31826892 | 0.03178203 |
| ABCB4 | 2.89505906 | 0.02915399 | 2.34545416 | 0.03005304 |
| LASS4 | 2.89041225 | 1.03370561 | 2.28112056 | 0.03429377 |
| VIP | 2.87181025 | 0.03487729 | 2.48674619 | 0.02239207 |
| PKHD1 | 2.87085337 | -2.1538102 | 2.39366365 | 0.02719991 |
| HOXD4 | 2.85982892 | -1.7967827 | 2.35002878 | 0.02977082 |
| CR2 | 2.85917993 | 0.03651799 | 2.10995291 | 0.04839967 |
| ZFP57 | 2.81882447 | -3.6032752 | 2.14296633 | 0.04532401 |
| SLC29A4 | 2.78916946 | 1.25386564 | 2.91755664 | 0.0088536 |
| SALL4 | 2.75513887 | 1.00103474 | 3.04259264 | 0.00671744 |
| GRB7 | 2.71713279 | 5.98905336 | 3.41497611 | 0.00291576 |
| UGT2B15 | 2.70580614 | 2.37227787 | 2.43773309 | 0.02481457 |
| OSTalpha | 2.69755232 | 0.39745724 | 2.09912838 | 0.04944863 |
| MEP1A | 2.69671479 | 4.76816881 | 2.39666344 | 0.02703099 |
| SSX4 | 2.68255697 | -0.8996802 | 2.17506483 | 0.04250523 |
| COL28A1 | 2.66390466 | -1.0122394 | 2.34936694 | 0.0298115 |
| MS4A12 | 2.54677144 | 0.82002213 | 2.11312647 | 0.04809598 |
| SNORA7B | 2.49759902 | -2.4472171 | 2.11671996 | 0.0477542 |
| UCN | 2.48369471 | -1.7779313 | 2.136213 | 0.04593832 |
| ZNF772 | 2.46492484 | -0.7596373 | 2.32287466 | 0.03148276 |
| SUZ12P | 2.42931644 | 2.18691366 | 2.13035503 | 0.04647731 |
| HOXD9 | 2.42489462 | 1.5181675 | 2.54731801 | 0.01970475 |
| ALDH3B2 | 2.41561667 | 0.04141898 | 2.51902148 | 0.02091999 |
| MARK1 | 2.40400098 | 0.22743379 | 2.63657011 | 0.01629365 |
| ABCB1 | 2.38672047 | 4.13131825 | 2.55479192 | 0.01939506 |
| LOC96610 | 2.30343159 | 8.63114019 | 2.16989148 | 0.04294844 |
| NELL2 | 2.25878482 | 0.03820171 | 2.62434752 | 0.01672528 |
| WNT5B | 2.2532563 | 1.71250618 | 2.22790285 | 0.03821206 |
| RARA | 2.24229411 | 5.55605194 | 2.98272813 | 0.00766925 |
| MOGAT3 | 2.22127843 | 2.92538609 | 2.26926786 | 0.03513295 |
| NINL | 2.19017214 | 1.57940713 | 2.25869645 | 0.03589735 |
| SYT7 | 2.16470518 | 4.44543701 | 2.35958692 | 0.02918907 |
| CYP2B6 | 2.12726651 | 3.48226838 | 2.10680996 | 0.04870214 |
| ERBB2 | 2.11793623 | 8.13403877 | 2.84942216 | 0.01027968 |
| SPTBN5 | 2.10471876 | 1.61341739 | 2.15166218 | 0.04454401 |
| ATG9B | 2.1020535 | 1.92181599 | 2.79892023 | 0.01147654 |
| GAS2 | 2.06287992 | -0.0231252 | 2.43212025 | 0.02510713 |
| ROBO2 | 2.04982603 | 1.23716516 | 2.31794702 | 0.03180304 |
| VGF | 2.01098217 | 0.40595675 | 2.17269314 | 0.0427079 |
| RPPH1 | 1.99862583 | 2.55995294 | 2.2829527 | 0.03416571 |
| PALM | 1.99045652 | 2.31745725 | 2.35735579 | 0.02932391 |
| LGR5 | 1.96597881 | 4.9233645 | 2.25921104 | 0.03585979 |
| C20orf46 | 1.94480925 | 2.09086834 | 2.64131642 | 0.0161289 |
| FAM101A | 1.90571417 | 4.66868028 | 2.49643415 | 0.02194028 |
| LOC613037 | 1.90347817 | 8.45784529 | 2.32139463 | 0.03157864 |
| KLC3 | 1.86850893 | 0.29036065 | 2.51931667 | 0.02090695 |
| NPIPL3 | 1.84463695 | 7.05249201 | 2.257748 | 0.03596668 |
| ACCS | 1.83572252 | 2.21897326 | 3.05442783 | 0.00654333 |
| GPER | 1.83536808 | 0.03418036 | 2.09976605 | 0.04938627 |
| KIFC2 | 1.83017519 | 4.22010336 | 2.57783736 | 0.0184688 |
| CAPN3 | 1.82954162 | 2.37476688 | 2.41176095 | 0.02619558 |
| PCSK4 | 1.82488268 | 0.97381674 | 2.40735184 | 0.02643703 |
| LOC100132247 | 1.82315753 | 8.67956335 | 2.29395463 | 0.03340598 |
| FOXH1 | 1.79039539 | -0.3478376 | 2.11309155 | 0.04809932 |
| CSAD | 1.7497254 | 2.53736858 | 2.77853056 | 0.01199666 |
| PTP4A3 | 1.7191467 | 5.22230634 | 3.15189938 | 0.00526676 |
| IGSF9 | 1.6706589 | 3.41828947 | 2.4035361 | 0.02664766 |
| CBX2 | 1.66500529 | 3.26283561 | 2.47264961 | 0.02306507 |
| EFHD1 | 1.66093208 | 0.71224212 | 2.25139825 | 0.03643397 |
| GDPD5 | 1.65325009 | 5.15164739 | 2.27376703 | 0.0348122 |
| KIAA1984 | 1.65069409 | 1.94999392 | 2.11464488 | 0.04795129 |
| ADAMTS13 | 1.64612942 | 1.17499873 | 2.10221672 | 0.04914727 |
| BCAM | 1.63329744 | 4.67563264 | 2.49883881 | 0.02182948 |
| LOC100132287 | 1.62193908 | 6.1884725 | 2.32286643 | 0.03148329 |
| HSF4 | 1.61084097 | 2.39303346 | 2.11526307 | 0.0478925 |
| PILRB | 1.60765226 | 5.08999289 | 2.25610985 | 0.0360867 |
| ZNF862 | 1.60568529 | 3.31596043 | 2.81911813 | 0.01098264 |
| TEAD2 | 1.60491819 | 4.46335795 | 3.22553279 | 0.00446673 |
| LOC100133331 | 1.59182518 | 5.19664403 | 2.30014318 | 0.03298552 |
| SRCIN1 | 1.56504594 | 2.80497429 | 2.31201634 | 0.03219251 |
| GALNTL4 | 1.56445374 | 3.27293691 | 2.2022211 | 0.04024649 |
| LOC100133161 | 1.56284232 | 5.11651914 | 2.22269747 | 0.03861663 |
| PDE1B | 1.56180567 | 0.11060208 | 2.15028085 | 0.04466709 |
| HLA-DQB1 | 1.5371472 | 5.82352837 | 2.20749582 | 0.03982071 |
| LOC338799 | 1.5309997 | 2.44605709 | 2.28162181 | 0.03425869 |
| EGR1 | 1.51918092 | 6.45401602 | 2.11181456 | 0.04822131 |
| HIST2H2BF | 1.51234231 | 1.46604697 | 2.82542653 | 0.01083261 |
| ProSAPiP1 | 1.51095735 | 4.88775747 | 2.86184238 | 0.01000423 |
| ZC3H12A | 1.49945888 | 5.32634375 | 2.28507424 | 0.03401798 |
| LDLRAD3 | 1.48209279 | 2.70531181 | 2.12394215 | 0.04707395 |
| ZNF337 | 1.47612109 | 3.70598296 | 2.52085481 | 0.02083916 |
| MAGED4B | 1.47106754 | 2.66519478 | 2.20672368 | 0.03988278 |
| GIGYF1 | 1.46863882 | 5.13094595 | 2.33675368 | 0.03059667 |
| ZFP36 | 1.45700608 | 6.73874062 | 2.58951585 | 0.01801552 |
| ZSCAN21 | 1.44440295 | 2.63213321 | 2.86424295 | 0.00995182 |
| MOSPD3 | 1.42098823 | 4.42331036 | 2.79746595 | 0.01151291 |
| RAB37 | 1.4206392 | 1.29799317 | 2.21407739 | 0.03929524 |
| DNASE1 | 1.38488202 | 3.04829855 | 2.14792428 | 0.04487778 |
| SLC12A9 | 1.37671743 | 5.92746988 | 2.26252078 | 0.03561907 |
| FJX1 | 1.3678375 | 2.40527162 | 2.26880708 | 0.03516596 |
| FUT1 | 1.36520019 | 2.83990229 | 2.30239081 | 0.03283403 |
| SLC26A11 | 1.33817459 | 2.87137407 | 2.56943877 | 0.01880142 |
| TCF7L1 | 1.33321063 | 2.10302104 | 2.11488741 | 0.04792822 |
| SLC6A6 | 1.32315206 | 5.81063951 | 2.71964795 | 0.01362829 |
| SCARB1 | 1.30590018 | 6.80158292 | 2.55242674 | 0.01949256 |
| ORAI2 | 1.30449441 | 4.42356274 | 2.33557001 | 0.03067132 |
| FAM113A | 1.29985704 | 4.4242691 | 2.53693134 | 0.02014288 |
| PRPF40B | 1.28690901 | 3.22971 | 2.14520459 | 0.04512206 |
| SHANK2 | 1.28253361 | 3.16386714 | 2.38071656 | 0.02794032 |
| GAA | 1.2816369 | 5.82773708 | 3.04241249 | 0.00672013 |
| ALS2CL | 1.27894764 | 4.56211131 | 2.55630784 | 0.01933281 |
| ACE | 1.25483091 | 4.99849566 | 2.18463614 | 0.04169623 |
| ELMO1 | 1.24852945 | 2.45226519 | 2.11969345 | 0.04747305 |
| SLCO2A1 | 1.24055879 | 3.57564909 | 2.10439257 | 0.04893595 |
| OR2A9P | 1.24042244 | 3.07725275 | 2.33254227 | 0.03086307 |
| TMEM132A | 1.21684415 | 5.26041257 | 2.40535611 | 0.026547 |
| ZNF623 | 1.21463928 | 3.71448361 | 3.16474046 | 0.00511783 |
| ZNF841 | 1.21226767 | 2.67746636 | 2.50814939 | 0.02140539 |
| ZNF696 | 1.20758292 | 3.24408485 | 2.3629261 | 0.02898833 |
| NCKAP5L | 1.20044499 | 3.29811061 | 2.16053545 | 0.04376072 |
| ZNF343 | 1.19790348 | 3.21368253 | 2.80820745 | 0.01124685 |
| GDF11 | 1.1962264 | 3.09563704 | 2.34982693 | 0.02978322 |
| TRRAP | 1.18975358 | 5.93377821 | 2.27561132 | 0.0346815 |
| ZFP41 | 1.18267194 | 3.6306985 | 2.09420239 | 0.04993277 |
| THBS3 | 1.17950605 | 3.89362067 | 2.7323838 | 0.01325836 |
| TNFAIP3 | 1.16722038 | 5.04339281 | 2.72436733 | 0.01349008 |
| TSC22D4 | 1.16143217 | 6.1243322 | 2.18020129 | 0.04206931 |
| JARID2 | 1.15512924 | 3.89629532 | 2.85423525 | 0.01017209 |
| PLXNB1 | 1.14038551 | 6.6766292 | 2.3398211 | 0.03040397 |
| SEMA4A | 1.13978552 | 3.20158142 | 2.3373354 | 0.03056004 |
| C11orf95 | 1.13433264 | 3.79599864 | 2.25420309 | 0.03622687 |
| EPHB4 | 1.13272535 | 7.1255205 | 2.2724855 | 0.03490329 |
| C20orf54 | 1.12964995 | 4.84540109 | 2.35027078 | 0.02975596 |
| TMEM79 | 1.1284205 | 2.71311895 | 2.34390299 | 0.0301493 |
| RALGAPA2 | 1.12362995 | 4.03681432 | 2.34321818 | 0.03019189 |
| ZNF517 | 1.11842841 | 3.38865261 | 2.31374737 | 0.03207838 |
| ZMYND8 | 1.11817431 | 5.64360073 | 2.61443319 | 0.01708333 |
| ZFP36L1 | 1.11096885 | 7.38776696 | 2.45995936 | 0.0236871 |
| IER5L | 1.09730325 | 4.55494293 | 2.42042432 | 0.02572714 |
| FAM110A | 1.09467078 | 4.26007785 | 2.60825682 | 0.01731004 |
| FAM156A | 1.08465137 | 5.93636437 | 2.14628489 | 0.04502488 |
| CEBPD | 1.07935777 | 5.47617976 | 3.12210639 | 0.00562876 |
| MGC12982 | 1.06372961 | 2.79306352 | 2.10401121 | 0.04897293 |
| VAT1 | 1.05276378 | 6.84468702 | 2.43444169 | 0.02498574 |
| C20orf96 | 1.04292848 | 3.23197042 | 2.43621363 | 0.02489345 |
| TMTC4 | 1.03325284 | 4.97084143 | 2.92610216 | 0.0086888 |
| OR2A7 | 1.02917776 | 4.05636661 | 2.40580062 | 0.02652247 |
| BAIAP2L1 | 1.01340403 | 7.00674661 | 2.34085415 | 0.03033933 |
| SLC39A10 | 1.01057479 | 4.59661995 | 2.51661843 | 0.02102638 |
| FOXP4 | 1.0075638 | 7.01519273 | 2.36076457 | 0.02911813 |
| CGN | 1.00717377 | 6.12784421 | 2.19996445 | 0.04042991 |
| ZNF133 | 1.00048019 | 3.86702216 | 2.41152121 | 0.02620866 |
| C6orf35 | -1.0022423 | 3.8640665 | -2.2546485 | 0.03619409 |
| MTERFD2 | -1.0046984 | 3.83628061 | -2.5049676 | 0.02154944 |
| S100A13 | -1.0059633 | 5.48693801 | -2.2346274 | 0.03769519 |
| SERF1A | -1.0090815 | 5.2850959 | -2.1079439 | 0.04859281 |
| CCNB2 | -1.0111849 | 5.60523545 | -2.1907955 | 0.04118309 |
| C15orf24 | -1.0172272 | 5.88512703 | -2.6217564 | 0.01681816 |
| INTS10 | -1.0277648 | 6.00946532 | -2.2491073 | 0.03660394 |
| ARHGEF10 | -1.0292816 | 3.73537602 | -2.4687276 | 0.02325566 |
| C10orf32 | -1.0312258 | 4.08819787 | -2.1298073 | 0.046528 |
| FHL2 | -1.0319187 | 7.23803643 | -2.1717476 | 0.04278894 |
| ARL 3.00 | -1.0323781 | 4.66556806 | -2.7239508 | 0.01350222 |
| PSMA4 | -1.0335 | 6.89028578 | -2.143796 | 0.04524905 |
| GSTO1 | -1.0377207 | 7.10642183 | -2.3339678 | 0.03077265 |
| ZDHHC6 | -1.0377529 | 5.27427183 | -2.3717038 | 0.02846676 |
| INPP1 | -1.0416539 | 5.00738158 | -3.0150141 | 0.00714078 |
| HFE | -1.0442524 | 2.59068502 | -2.3343668 | 0.03074739 |
| PRDX3 | -1.0449837 | 7.53209005 | -2.4847921 | 0.02248425 |
| ELL3 | -1.0461455 | 3.86740045 | -2.5318399 | 0.02036099 |
| IDH2 | -1.0497518 | 8.00508987 | -2.1373451 | 0.04583481 |
| ERCC8 | -1.0602037 | 2.80122331 | -2.2709465 | 0.03501297 |
| GMNN | -1.0667122 | 4.82236221 | -2.2596752 | 0.03582594 |
| CNOT7 | -1.067557 | 5.92898504 | -2.1884493 | 0.04137787 |
| RPS27L | -1.0681183 | 5.60572269 | -2.6052376 | 0.0174219 |
| GJB3 | -1.0716886 | 5.33718342 | -2.3716261 | 0.02847134 |
| C18orf55 | -1.0728733 | 4.04598157 | -2.2534354 | 0.03628345 |
| C1QBP | -1.073064 | 7.8191903 | -2.7231112 | 0.01352673 |
| TRNAU1AP | -1.0750243 | 4.25974811 | -2.7800994 | 0.01195586 |
| MTHFD2 | -1.0875305 | 6.22174695 | -2.2346995 | 0.03768967 |
| RSL24D1 | -1.0934095 | 6.58925159 | -2.1219756 | 0.0472583 |
| PTTG1 | -1.096554 | 5.23693499 | -2.6721983 | 0.01509498 |
| MRPS22 | -1.0974605 | 5.35899657 | -2.6572651 | 0.01558682 |
| COX7A2 | -1.0984831 | 7.66397543 | -2.2733994 | 0.03483831 |
| CD47 | -1.0987514 | 6.5857505 | -2.4948349 | 0.02201427 |
| GFM2 | -1.1001576 | 4.47084201 | -2.4275151 | 0.02534957 |
| RPRD1A | -1.1011014 | 5.06678282 | -2.42689 | 0.02538265 |
| MRPS35 | -1.1030594 | 6.7340958 | -2.8783244 | 0.00964964 |
| PSMA6 | -1.1036342 | 7.25944031 | -2.3563562 | 0.02938451 |
| PIAS2 | -1.1052114 | 2.79628761 | -2.1293145 | 0.04657365 |
| ANXA2P2 | -1.1062175 | 9.03120784 | -2.5003504 | 0.0217601 |
| CYB5A | -1.1069011 | 6.18284774 | -2.483402 | 0.02255004 |
| LRP8 | -1.1121463 | 3.82971091 | -2.3178243 | 0.03181105 |
| TXNL1 | -1.1180839 | 5.91336005 | -2.5079947 | 0.02141238 |
| TXNDC17 | -1.1231659 | 6.2738633 | -2.9291513 | 0.00863072 |
| C10orf84 | -1.1256674 | 4.47728429 | -2.1088236 | 0.04850815 |
| TOMM22 | -1.1257826 | 6.59023943 | -2.2240853 | 0.03850838 |
| BOLA3 | -1.1282707 | 4.4855384 | -2.612752 | 0.01714476 |
| PPA2 | -1.1301779 | 6.29033057 | -2.6106808 | 0.01722073 |
| ZNF511 | -1.1343427 | 5.8986332 | -2.1513159 | 0.04457483 |
| DEPDC1B | -1.1371734 | 3.26774473 | -2.1707884 | 0.0428713 |
| ANXA2 | -1.1382092 | 10.1462743 | -2.4677512 | 0.02330334 |
| SH2D4A | -1.1397489 | 4.62787219 | -2.5189486 | 0.02092321 |
| RRM2 | -1.1418992 | 6.48090067 | -2.3118773 | 0.03220169 |
| C15orf63 | -1.1425646 | 6.35111345 | -2.2812354 | 0.03428573 |
| C1orf151 | -1.1469297 | 6.62300712 | -2.7486 | 0.0128012 |
| FAM96A | -1.1527824 | 5.6713922 | -2.5875538 | 0.01809093 |
| MRPS36 | -1.153026 | 4.22224437 | -2.7186836 | 0.0136567 |
| C14orf167 | -1.162033 | 4.05547495 | -2.5658421 | 0.01894558 |
| ZG16B | -1.1735718 | 5.21079725 | -2.2453367 | 0.03688527 |
| CAPG | -1.1750058 | 7.4344966 | -2.2248837 | 0.03844624 |
| SPNS2 | -1.1776956 | 5.77619616 | -2.3669934 | 0.02874556 |
| GHITM | -1.181527 | 8.00257697 | -2.5260147 | 0.02061325 |
| SNRNP25 | -1.1887279 | 5.54172593 | -2.2049579 | 0.04002506 |
| DYRK4 | -1.1888365 | 4.46267137 | -2.1390562 | 0.04567877 |
| CISD2 | -1.1922007 | 5.25671646 | -2.9554112 | 0.00814577 |
| PDE4C | -1.1932445 | 2.86623918 | -2.5410795 | 0.01996682 |
| APOO | -1.1968518 | 3.6277205 | -2.6997493 | 0.01422603 |
| GSR | -1.2001149 | 6.29021209 | -2.2286498 | 0.03815433 |
| PYROXD1 | -1.200483 | 4.04260059 | -2.1924326 | 0.04104768 |
| EPHX4 | -1.2056531 | 3.31852406 | -2.5290858 | 0.02047989 |
| TBCA | -1.2150813 | 5.97869034 | -2.9030744 | 0.0091398 |
| ARG2 | -1.2192517 | 3.96013973 | -2.6313554 | 0.01647649 |
| GLRX | -1.2200348 | 5.67622972 | -2.1778836 | 0.0422655 |
| SKA1 | -1.22046 | 3.26271435 | -2.4203078 | 0.02573339 |
| TNFSF10 | -1.2214502 | 5.91915295 | -2.3730493 | 0.02838759 |
| AKR7L | -1.235207 | 3.49080412 | -2.53196 | 0.02035582 |
| SNRPG | -1.2362532 | 6.38171349 | -2.4178178 | 0.02586725 |
| CCNA2 | -1.2416364 | 5.38315483 | -2.8330922 | 0.01065294 |
| EPR1 | -1.2469954 | 5.82329042 | -2.701893 | 0.01416046 |
| MAD2L1 | -1.2527427 | 5.23181589 | -2.2393261 | 0.03733784 |
| NDUFB1 | -1.2544332 | 5.46261525 | -2.2763215 | 0.03463129 |
| MOCS2 | -1.2687561 | 4.47254744 | -2.344275 | 0.03012619 |
| CKLF | -1.2706837 | 5.42022511 | -2.705536 | 0.01404968 |
| S100A3 | -1.2744522 | 1.10779416 | -2.1816126 | 0.04195026 |
| C15orf29 | -1.2849739 | 3.30526087 | -2.1026869 | 0.04910153 |
| LXN | -1.2930795 | 4.02190101 | -2.6806247 | 0.01482399 |
| CENPW | -1.3072024 | 4.90903178 | -2.6312393 | 0.01648059 |
| LOC1001309 | -1.318222 | 4.94157259 | -2.4353185 | 0.02494004 |
| NDUFS4 | -1.32083 | 5.24778051 | -3.2303655 | 0.00441859 |
| C10orf78 | -1.3294368 | 2.88422892 | -2.6396835 | 0.0161854 |
| ANXA2P1 | -1.3297587 | 4.82625179 | -3.056026 | 0.00652015 |
| GJB4 | -1.3499832 | 3.14079135 | -2.4667925 | 0.02335024 |
| CDKN3 | -1.3569278 | 4.14340589 | -2.2575167 | 0.0359836 |
| GLRX3 | -1.3584988 | 6.24800958 | -3.175192 | 0.00499965 |
| PYGL | -1.3605024 | 5.22496848 | -2.5936954 | 0.01785587 |
| CDCA2 | -1.3781608 | 2.71132583 | -2.2580267 | 0.03594629 |
| PIR | -1.4013491 | 3.47166124 | -2.4680331 | 0.02328956 |
| NDUFAF2 | -1.4057407 | 4.01831126 | -3.2290081 | 0.00443206 |
| LOC81691 | -1.4074486 | 2.11813193 | -2.6139177 | 0.01710214 |
| CCNB1 | -1.4116079 | 6.4651729 | -3.293695 | 0.00383279 |
| CKS2 | -1.4152546 | 6.13826806 | -2.3275647 | 0.0311807 |
| KIAA0101 | -1.4269933 | 4.51912286 | -2.3940347 | 0.02717897 |
| HES6 | -1.4560623 | 5.79166051 | -2.1982827 | 0.04056711 |
| FAM81A | -1.4625477 | 2.57845436 | -2.5432963 | 0.01987332 |
| INPP5D | -1.466551 | 4.96218296 | -3.0485205 | 0.00662968 |
| ASS1 | -1.4793082 | 8.78816975 | -3.468742 | 0.00258186 |
| GPR114 | -1.4920427 | 2.39495954 | -2.5814001 | 0.01832939 |
| CMBL | -1.5407666 | 5.8013159 | -2.4569805 | 0.02383538 |
| BIK | -1.5696544 | 4.20542952 | -2.8577082 | 0.01009512 |
| ATPIF1 | -1.5988013 | 7.1256877 | -3.8833676 | 0.00100557 |
| CENPK | -1.6012682 | 2.67589919 | -2.6058277 | 0.01739999 |
| WDR76 | -1.6026317 | 3.08243683 | -2.9774565 | 0.00775906 |
| MT1E | -1.6047682 | 4.37888904 | -2.262235 | 0.0356398 |
| CCL4 | -1.6288755 | 2.41592285 | -2.1518718 | 0.04452536 |
| S100P | -1.6385583 | 8.69119233 | -2.2169783 | 0.03906566 |
| DHFR | -1.6455987 | 5.45342562 | -3.4014515 | 0.00300624 |
| NQO1 | -1.6520799 | 7.06785866 | -3.106181 | 0.00583208 |
| CTSW | -1.653702 | 1.13454031 | -2.4352583 | 0.02494317 |
| MLF1IP | -1.661584 | 4.46071996 | -2.6875315 | 0.01460533 |
| ANKRD22 | -1.6646614 | 5.54899822 | -3.7915787 | 0.00123961 |
| PBK | -1.705044 | 4.08838528 | -2.2910966 | 0.03360182 |
| ESCO2 | -1.7213592 | 2.8645732 | -2.2878785 | 0.03382362 |
| CCL3 | -1.7541006 | 2.62363319 | -2.2413944 | 0.03718154 |
| AKR7A3 | -1.761095 | 5.52279185 | -2.5114712 | 0.02125597 |
| FLJ46111 | -1.770519 | 0.52930189 | -2.2791534 | 0.03443175 |
| CCDC108 | -1.777428 | -0.4516695 | -2.1305282 | 0.04646129 |
| TTC32 | -1.8371671 | 1.02449908 | -2.31368 | 0.03208282 |
| ZNF655 | -1.8852045 | 3.9842478 | -3.4903362 | 0.00245863 |
| GALNT5 | -1.8969536 | 4.0615574 | -2.9329063 | 0.00855971 |
| CHAC2 | -1.9139092 | 1.62598749 | -2.5012701 | 0.02171798 |
| PTPRN2 | -1.9391374 | 5.24747108 | -2.5641606 | 0.01901334 |
| KLK10 | -1.941203 | 4.43807834 | -2.8010933 | 0.0114224 |
| C1orf183 | -1.9601684 | 0.39961673 | -2.7041847 | 0.01409067 |
| C4orf36 | -1.9618268 | -1.0991803 | -2.328774 | 0.03110325 |
| ZNF544 | -1.978523 | 3.4878964 | -3.4445967 | 0.00272687 |
| GSTO2 | -1.9978037 | 4.02856803 | -3.0386398 | 0.00677659 |
| AKAP3 | -2.0088245 | -0.4592294 | -2.162698 | 0.04357174 |
| PIWIL2 | -2.0617686 | 0.4168955 | -2.6202793 | 0.01687133 |
| IL1RN | -2.0666629 | 3.56373228 | -3.1334534 | 0.00548812 |
| UBXN10 | -2.0801025 | 3.23332539 | -3.6820914 | 0.00159063 |
| B3GALNT1 | -2.1268412 | 1.62443836 | -2.8502964 | 0.01026006 |
| SHF | -2.1664498 | 3.65312554 | -2.7050247 | 0.01406517 |
| ZNF702P | -2.1731864 | 1.7924595 | -2.5917263 | 0.01793092 |
| FAM131B | -2.1950932 | 2.80093741 | -2.508375 | 0.02139521 |
| FGGY | -2.2043193 | 4.68409387 | -2.2171659 | 0.03905085 |
| LDHB | -2.2147232 | 7.67213164 | -2.8472755 | 0.01032802 |
| AK5 | -2.2424628 | -1.7435267 | -2.2133264 | 0.03935487 |
| LEMD1 | -2.2579345 | 1.13161818 | -2.1983055 | 0.04056525 |
| C12orf60 | -2.3341162 | -1.1517516 | -2.2032027 | 0.04016694 |
| HS6ST2 | -2.3634248 | 2.27960779 | -2.5066198 | 0.02147453 |
| MESP2 | -2.3747498 | -1.0703176 | -2.1937688 | 0.04093745 |
| IL8 | -2.3840536 | 5.37833905 | -2.7430636 | 0.01295556 |
| LYPD5 | -2.4102085 | 1.76423376 | -2.7384087 | 0.01308672 |
| C1orf229 | -2.6440661 | -2.0600069 | -2.2059794 | 0.03994269 |
| LOC100129716 | -2.6692581 | -1.2312837 | -2.5066987 | 0.02147096 |
| FAM19A1 | -2.6935062 | -1.1523153 | -2.5023945 | 0.0216666 |
| HESRG | -2.7197349 | 0.22014409 | -2.4398458 | 0.02470528 |
| DSG3 | -2.8161671 | 2.39335181 | -3.2269636 | 0.00445243 |
| LYZ | -2.9474651 | 8.79847496 | -2.3993173 | 0.02688236 |
| IRX3 | -3.1349192 | -1.2114567 | -2.5677962 | 0.01886713 |
| KLK7 | -3.1995927 | -0.4817412 | -2.1609719 | 0.04372252 |
| LOC647946 | -3.2716981 | -1.1645286 | -2.5497982 | 0.01960147 |
| C14orf34 | -3.3347614 | -2.3493479 | -2.3799796 | 0.02798302 |
| DNAH9 | -3.5270835 | -2.2368787 | -2.6216712 | 0.01682122 |
| PLA2G3 | -3.7054812 | -1.1450493 | -2.8266719 | 0.01080322 |
| C5orf38 | -3.8387564 | -0.9391674 | -2.764446 | 0.01236904 |
| FIBCD1 | -4.17766 | 1.75351992 | -3.4655775 | 0.00260042 |
| NPSR1 | -4.675893 | 0.62622433 | -2.175961 | 0.04242888 |

Table S6. CXCL1 transcription factor prediction and MAGEA6 interacting protein

| **Transcription factor** | | **MAGEA6 interacting protein** | |
| --- | --- | --- | --- |
| **Signaling pathways** | **ChIP-****Atlas** | **HCT116** | **HT29** |
| FOSL2 | AGO2 | XRCC6 | NTHL1 |
| SRF | AHR | RBMX | RBM25 |
| E2F3 | ARID2 | FLG | SLC2A1 |
| ELF1 | ARID5B | KPRP | ZFP91 |
| TP73 | ATRX | SDAD1 | ALB |
| TBP | BCLAF1 | ALDOB | H1-2 |
| UBTF | BRD7 | MRPS18A | NCOA5 |
| YY1 | CBFA2T3 | FUS | MRPS28 |
| HEY1 | CBFB | NXF1 | NOL10 |
| STAT3 | CBX3 | RPS5 | MRPL15 |
| USF1 | CDK9 | DARS1 | CEP112 |
| EGR1 | CDX2 | PRKDC | RPN2 |
| SATB1 | CEBPA | POF1B | IST1 |
| FOXA1 | CEBPB | HNRNPU | H4C16 |
| FOXP1 | CHD1 | RCC2 | TUBB4B |
| CTCF | CTCF | SFXN1 | APOE |
| CEBPA | CTNNB1 | SPATS2L | YBX1 |
| PBX3 | DAXX | UPF3B | NOL6 |
| TP53 | DDX5 | CEBPZ | UQCRC2 |
| GATA2 | DNMT1 | NCCRP1 | SNRPE |
| ATF2 | DPF2 | RNPS1 | UTP15 |
| ELF3 | EZH2 | GRWD1 | INO80 |
| ERG\|DHT | FUS | CHD2 | DDX47 |
| KLF11 | GATAD2A | FARSA | NUP93 |
| KLF5 | GATAD2B | NHP2 | SYNCRIP |
| MAFF | GLYR1 | SRSF10 | RPS27 |
| SP1 | HDAC1 | TUFM | TRIP13 |
| CREB1 | HDAC2 | PPAN | ZNF512 |
| HOXA4 | HMGN1 | DHX9 | HNRNPDL |
| MAZ | HNRNPL | CHTOP | ALDOB |
| JUN | HNRNPLL | SLC25A22 | MBD2 |
| CEBPB | JUNB | HNRNPR | RAB7A |
| HMGN3 | KAT7 | STRAP | LACTB |
| TEAD1 | KDM1A | TRA2B | PKP3 |
| GABPA | KDM2B | RPL39P5 | CYC1 |
| GATA4 | KMT2A | NOLC1 | CDK1 |
| IRF1 | KMT2D | MYO1C | NOP56 |
| LYL1 | LARP7 | PURA | SUB1 |
| TCF7L2 | LIG4 | CLTC | KIF2A |
| E2F6 | LMNA | CALM3 | PDCD11 |
| FOXA2 | LMNB1 | SSR1 | TUBA1B |
| STAT1\|IFNG | MAFG | RTCB | EFTUD2 |
| MYC | MAFK | C11orf54 | CCDC86 |
| TFAP4 | MAPK1 | YTHDC1 | FAM91A1 |
| REST | MYH11 | PRC1 | LSM14B |
| TEAD4 | NONO | HMGN1 | SRSF6 |
| RELA | PARK7 | EIF2S3 | CMSS1 |
| ZNF143 | PHF8 | SPOUT1 | EWSR1 |
| IRF1\|IFNG | POLR2A | NMNAT1 | FXR1 |
| RUNX1 | PPARG | DHX33 | RPL35 |
| TCF21 | RBBP7 | PRPF6 | ATXN2 |
| FOS | RBM25 | FIP1L1 | CASZ1 |
| ZBTB7A | REST | RPA2 | SF3B3 |
| MAX | RNF2 | HSP90AB2P | VAV1 |
| SMAD1 | RUNX1 | TCF20 | PPAN |
| JUND | SIN3A | MYL6 | CPS1 |
|  | SMARCA4 | HNRNPA3 | ACADM |
|  | SMARCB1 | POP1 | SF3B2 |
|  | SMARCC1 | DSG1 | TRIM29 |
|  | SMARCC2 | TMPO | SFXN1 |
|  | SMC1A | GSTA2 | RPN1 |
|  | SMC3 | XRCC1 | C6orf132 |
|  | SMC4 | MRPL15 | SMU1 |
|  | SMCHD1 | JCHAIN | CES1 |
|  | STAT1 | HNRNPDL | POF1B |
|  | SUPT20H | RTRAF | LCN1 |
|  | SUZ12 | RRP1 | SF3B1 |
|  | TCF7L2 | MRPL18 | PCBP1 |
|  | TDRD3 | ASPH | AFG3L2 |
|  | TFAP4 | KIF22 | ZFC3H1 |
|  | THAP11 | CPS1 | AP2M1 |
|  | TP53 | MARK2 | PCBD1 |
|  | TRIM25 | CDK9 | HSPE1 |
|  | TRIM28 | CHMP2B | RSL1D1 |
|  | TRRAP | NOP2 | STK39 |
|  | UBTF | HSPE1 | DDX28 |
|  | USP7 | CHMP1A | TUFM |
|  | WDR5 | ELL3 | FMR1 |
|  | XRCC4 | EIF3I | UBAP2L |
|  | YY1 | MYBBP1A | HNRNPAB |
|  | 5-mC | CDK1 | ILF2 |
|  | AFF4 | IARS1 | STT3A |
|  | AML1-ETO | TOMM22 | SPATS2 |
|  | AR | ANXA7 | PKP2 |
|  | ARID1A | MKI67 | TRA2B |
|  | ARID1B | FXR2 | CYB5A |
|  | ARNTL | ALB | CSNK1G1 |
|  | ASCL1 | CHCHD3 | FXR2 |
|  | ASXL1 | PSMA5 | MAP7 |
|  | ATF2 | RARS1 | COPA |
|  | ATF3 | SF3A1 | DHX9 |
|  | ATF4 | RPF2 | MYO5C |
|  | BACH2 | PPP2R2A | MCM7 |
|  | BANP | POLR1G | LCOR |
|  | BATF | FAM98A | NHP2 |
|  | BCL11A | DDX21 | GCDH |
|  | BCL3 | RBM28 | SF3A2 |
|  | BCL6 | PXK | IQGAP1 |
|  | BCOR | SMARCE1 | LGALS7B |
|  | BDP1 | CTSD | YBX3 |
|  | BHLHE40 | MYH9 | CLTC |
|  | Biotin | DR1 | AP3B1 |
|  | BPTF | ERLIN2 | MPHOSPH10 |
|  | BRCA1 | EIF2S2 | CCDC137 |
|  | BRD2 | RPS10 | ABT1 |
|  | BRD3 | MRPL38 | RCL1 |
|  | BRD4 | MAGT1 | CKAP5 |
|  | BRF1 | SRRM2 | SLX9 |
|  | BRF2 | S100A9 | CYFIP1 |
|  | C17orf49 | EIF6 | TCF20 |
|  | CARM1 | RACGAP1 | TUBA4A |
|  | CBX7 | PCBD1 | PLCD3 |
|  | CCDC101 | STAU1 | PRPF4 |
|  | CDK6 | BMS1 | TASOR |
|  | CDK8 | RPL35 | RRP1B |
|  | CDKN1B | GTPBP10 | HARS2 |
|  | CDYL2 | CES1 | YTHDF2 |
|  | CEBPD | ABCF1 | RPF2 |
|  | CHAT | SRSF3 | TAF6L |
|  | CHD8 | SNRPN | ILF3 |
|  | CIC | ZNF512 | C11orf98 |
|  | CLOCK | GSN | PRRC2C |
|  | CREB1 | CSNK1D | SRRM2 |
|  | CREBBP | YME1L1 | EIF6 |
|  | CRTC2 | HNRNPD | HMG20A |
|  | CSF1R | RRP15 | H1-4 |
|  | CTCFL | FXR1 | IGHG3 |
|  | CXXC1 | DDX18 | PRRC2A |
|  | DCP1A | CASZ1 | CHMP3 |
|  | DDIT3 | SLC25A5 | LSG1 |
|  | DOT1L | RPL29 | ABCE1 |
|  | DUX4 | RPL32 | CTCF |
|  | E2F1 | LGALS3 | CHMP5 |
|  | E2F4 | ARG1 | H1-0 |
|  | E2F6 | H1-10 | PLEKHA5 |
|  | E2F7 | HRNR | IMP4 |
|  | EBF1 | IGLV2-8 | IGF2BP2 |
|  | EBNA2 | RRBP1 | CSNK1D |
|  | EGR1 | SLC16A1 | NOP16 |
|  | EGR2 | GTPBP4 | SLC25A5 |
|  | ELF1 | WDR12 | GLYR1 |
|  | ELF3 | RPL4 | DDX49 |
|  | ELK1 | CAPRIN1 | SON |
|  | ELK4 | FLG2 | CHMP4B |
|  | ELL2 | MCM3 | IGF2BP3 |
|  | EOMES | DDX41 | SMARCE1 |
|  | EP300 | USP39 | NCL |
|  | EP400 | HBA2 | RRS1 |
|  | ERG | LIMA1 | DHX30 |
|  | ESR1 | BAZ1A | SLC25A24 |
|  | ESR2 | PGAM5 | DHX15 |
|  | ETS1 | APOE | RPS8 |
|  | ETV1 | SLC25A12 | RRP7A |
|  | ETV4 | KIFC1 | AATF |
|  | FGFR1 | TXN | HOXB9 |
|  | FLI1 | APRT | CAMK2D |
|  | FOS | KIF2A | KIFC1 |
|  | FOSL1 | RBM14 | NOP2 |
|  | FOSL2 | C11orf98 | IGHA1 |
|  | FOXA1 | MRPL11 | SF3A1 |
|  | FOXA2 | SRSF7 | IGLV2-8 |
|  | FOXH1 | NOC4L | SF1 |
|  | FOXJ3 | TRIM56 | APTX |
|  | FOXL2 | SRSF9 | PURA |
|  | FOXO1 | UTP3 | CKAP4 |
|  | FOXP1 | FABP5 | FAM120A |
|  | FOXP2 | JUND;JUN | SLC25A3 |
|  | GABPA | MPHOSPH10 | SLC25A6 |
|  | GATA1 | IGHG1 | NOC4L |
|  | GATA2 | SMARCAL1 | EIF2S3 |
|  | GATA3 | RGN | H1-10 |
|  | GATA4 | NOB1 | NPM1 |
|  | GATA6 | AATF | ESYT2 |
|  | GATAD1 | ITIH2 | CYCS |
|  | GFP | EIF2AK2 | ATAD3B |
|  | GMEB2 | NPM1 | HOXA5 |
|  | GRHL1 | UTP11 | POLR1G |
|  | HAP1 | PPP1CA | LIMA1 |
|  | HCFC1 | LTF | IK |
|  | HDAC3 | LSM14B | RBM34 |
|  | HDAC6 | GAPDH | TMEM33 |
|  | HDAC8 | PSMA1 | RPL29 |
|  | HEXIM1 | DNAJA3 | SLC25A22 |
|  | HEY1 | DDX31 | IGKC |
|  | HIF1A | HNRNPA1 | GRWD1 |
|  | HIV Tat | ARPC2 | CHMP2B |
|  | HNF1A | CYB5A | RPS11 |
|  | HNF4A | HARS2 | SRSF7 |
|  | HNF4G | SART1 | TRA2A |
|  | HOXB13 | PWP1 | GNMT |
|  | HOXC5 | AZGP1 | TAF5L |
|  | HSF1 | MAFK | CHD1L |
|  | IKZF1 | RPL12 | SSR1 |
|  | INTS12 | HNRNPAB | SDF4 |
|  | INTS13 | DDX47 | HNRNPR |
|  | IRF1 | DDX54 | HNRNPA0 |
|  | IRF2 | ALDH1A3 | REXO4 |
|  | IRF4 | COX5A | MRTO4 |
|  | JARID2 | ZNF638 | AP1M2 |
|  | JMJD1C | SLC16A3 | DDX18 |
|  | JUN | MTREX | FAU |
|  | JUND | NAP1L1 | RPL26 |
|  | KAT2B | RPL6 | PRKRA |
|  | KAT5 | RPL19 | MRPL16 |
|  | KAT8 | MRM1 | FBXW11 |
|  | KDM2A | RPS8 | HOXA13 |
|  | KDM4A | ATXN2L | EMD |
|  | KDM5B | RRP12 | MAP7D1 |
|  | KLF1 | TBL2 | SMARCA5 |
|  | KLF3 | TOR4A | DDX21 |
|  | KLF4 | NUSAP1 | HMGB1 |
|  | KLF6 | DNAJC9 | RPL6 |
|  | L3MBTL2 | GNMT | RPS3 |
|  | LDB1 | SLC25A6 | RACGAP1 |
|  | LEO1 | SGPL1 | DDX50 |
|  | LIN9 | DSC3 | ABCF1 |
|  | LMO2 | CAMK2D | RBM28 |
|  | LMTK3 | LARP7 | VRK3 |
|  | LRIF1 | RPL3 | RPL13A |
|  | LRWD1 | HP1BP3 | MRPS23 |
|  | LYL1 | RRP1B | RPL19 |
|  | MAML3 | AGK | PRC1 |
|  | MAX | TOP2B | DNAJA2 |
|  | MAZ | NOL10 | RHBDF1 |
|  | MBD3 | XRN2 | ARHGAP32 |
|  | MCPV ST | KDM1A | PES1 |
|  | MED1 | SRPK1 | FTSJ3 |
|  | MED12 | G3BP1 | EBNA1BP2 |
|  | MED26 | ATAD3B | DDX23 |
|  | MEF2A | CYCS | ACTR2 |
|  | MEF2B | PRKRA | DNAJA1 |
|  | MEF2C | RPL5 | DKC1 |
|  | MEIS1 | PTDSS1 | ZC3HAV1 |
|  | MethylCap | DDT;DDTL | PRPF8 |
|  | MLL-AF6 | PRPF19 | RPS2 |
|  | MLLT3 | LRRC59 | NOL9 |
|  | MXI1 | BRIX1 | RPL21 |
|  | MYB | NUP93 | RPS4X |
|  | MYBL2 | SPATA5 | SNRPN |
|  | MYC | PSIP1 | MAGT1 |
|  | MYCN | POLDIP3 | RPL13 |
|  | NANOG | YTHDC2 | BCAS2 |
|  | NCOA1 | DDX49 | THAP11 |
|  | NCOA3 | PSMC1 | SART1 |
|  | NCOR1 | TMEM33 | RPLP0 |
|  | NCOR2 | CCDC137 | SNRNP40 |
|  | NELFA | YTHDF2 | KIF23 |
|  | NELFCD | RPLP0 | DDX41 |
|  | NELFE | SLC25A1 | MRM3 |
|  | NEUROD1 | MACROH2A2 | RPF1 |
|  | NFATC1 | C7orf50 | BMS1 |
|  | NFE2 | AURKB | MYO1E |
|  | NFKBIA | CSNK2B | RPL10A |
|  | NFYA | A2ML1 | DNAJC9 |
|  | NFYB | H1-0 | SRPK1 |
|  | NKX2-1 | RPL14 | RPL8 |
|  | NOTCH1 | CEP170 | KIAA1522 |
|  | NR1H3 | SNRNP40 | NIFK |
|  | NR2C2 | CPNE3 | BRD1 |
|  | NR2F1 | ABCF2 | RRP1 |
|  | NR2F2 | HACD3 | RPUSD4 |
|  | NR3C1 | KIF23 | DDX52 |
|  | NR4A1 | RPS4X | ETFB |
|  | NR5A1 | CKAP4 | RPL11 |
|  | NRIP1 | BOP1 | NSA2 |
|  | O-GlcNAc | DCAF13 | RBM17 |
|  | ONECUT2 | GNL3 | RPL4 |
|  | ORC2 | EMD | THOC5 |
|  | PAF1 | ALKBH5 | RBM39 |
|  | PALB2 | CYC1 | PRPF6 |
|  | PAX5 | PRDX5 | NOP14 |
|  | PAX8 | IGKC | RPL7A |
|  | PBX3 | WDR36 | XRN2 |
|  | PBX4 | LUC7L | ABCD3 |
|  | PBXIP1 | RPL7A | USP10 |
|  | PCGF1 | RPS6 | THOC1 |
|  | pFM2 | MRM3 | PRPF31 |
|  | PIAS1 | SLC25A11 | STON2 |
|  | PML | FTSJ3 | RPL14 |
|  | POLR2C | RPS15 | SQOR |
|  | POU2F1 | SLC25A3 | C8orf33 |
|  | POU2F2 | CIRBP | PRPF3 |
|  | POU5F1 | EXOSC9 | MTPAP |
|  | PRAME | RPL13A | EIF2B4 |
|  | PRDM1 | RPL13 | SDAD1 |
|  | PRDM10 | RPL7 | PUM3 |
|  | PRDM14 | NAT10 | MRPL11 |
|  | PTEN | BCAS2 | H1-3 |
|  | RAD21 | DECR1 | RPS3A |
|  | RAD51 | MYO6 | EIF2S2 |
|  | RAG1 | PRDX1 | LUC7L3 |
|  | RAG2 | HMGA1 | DECR1 |
|  | RARA | HBB | DNAJA3 |
|  | RB1 | SRP72 | ITIH2 |
|  | RBFOX2 | ERLIN1 | RALY |
|  | RBL1 | CBX8 | PHF14 |
|  | RBL2 | TCF7L2 | XPC |
|  | RBPJ | RBM12B | HP1BP3 |
|  | RELA | LARP4B | PRPF19 |
|  | RFX5 | RPS3 | DPM1 |
|  | RUNX1T1 | TFAM | GNL3 |
|  | RUNX2 | DNAJA2 | DDX56 |
|  | RUNX3 | CPA4 | SRP72 |
|  | RXRA | BHMT | DDX54 |
|  | SATB1 | DCD | SLC25A11 |
|  | SETD1A | ALYREF | RPL10 |
|  | SETDB1 | MYL12A | RPL17 |
|  | SIX5 | TIMM50 | ADAR |
|  | SKI | RPL8 | ACTR3 |
|  | SMAD1 | UTP15 | MRPS7 |
|  | SMAD2 | SNRPB2 | NOC3L |
|  | SMAD3 | RPL10A | XPO1 |
|  | SMAD4 | LCN2 | RGN |
|  | SNAI2 | RPS3A | ATXN2L |
|  | SNAPC1 | ACTA1 | S100A11 |
|  | SNAPC2 | ZNF384 | DNAJB11 |
|  | SOX2 | PIP | LSM12 |
|  | SP1 | PHC2 | ESF1 |
|  | SP2 | CALML5 | RPS6 |
|  | SPI1 | BYSL | RRP15 |
|  | SREBF2 | NIFK | TBL2 |
|  | SRF | HNRNPH2 | LTF |
|  | STAG1 | IGKV2-29 | RPL7 |
|  | STAG2 | HNRNPA0 | SMARCA1 |
|  | STAT3 | NKRF | AP3D1 |
|  | STAT4 | TEX10 | RPS9 |
|  | STAT5A | PTCD3 | SNRNP200 |
|  | STAT5B | GAR1 | DDX27 |
|  | SUMO2 | ZNF629 | RBM8A |
|  | SUPT5H | RPL26 | RPL5 |
|  | TAF1 | ATAD3A | RPS7 |
|  | TAF2 | EIF2S1 | RNPS1 |
|  | TAF3 | ADAR | ACSL5 |
|  | TAF7 | RFC2 | SPATS2L |
|  | TAL1 | CBX3 | NAP1L1 |
|  | TBP | IGHA1;IGHA2 | RPL3 |
|  | TBX21 | DHX30 | NSUN2 |
|  | TCF12 | CBX5 | MRM1 |
|  | TCF3 | MRPS22 | SSB |
|  | TCF4 | RPS9 | TCF7L2 |
|  | TEAD1 | SURF6 | LIG3 |
|  | TEAD4 | KARS1 | NIP7 |
|  | TET2 | GNL2 | PNN |
|  | TFAP2A | RPS7 | RPL23A |
|  | TFAP2C | RPL11 | MPHOSPH8 |
|  | THAP1 | TOP1 | POLR2B |
|  | TLE3 | RBM7 | PGAM5 |
|  | TP53BP1 | NOP16 | NOLC1 |
|  | TP63 | RBM8A | EXOSC9 |
|  | TP73 | SRP68 | BRIX1 |
|  | TRIM24 | NSA2 | KRI1 |
|  | TRP47 | RPL10 | H1-5 |
|  | USF1 | DDX55 | TECR |
|  | USF2 | MRPS18B | RRP8 |
|  | VDR | RPL17 | AGK |
|  | WRN | MYO1D | ASPH |
|  | WWTR1 | RPS2 | EIF2S1 |
|  | YAP1 | PPP1CC | SNRPB2 |
|  | ZBTB16 | RFC3 | LARP1 |
|  | ZBTB33 | RNF2 | HACD3 |
|  | ZBTB7A | NOC3L | YME1L1 |
|  | ZEB1 | NOM1 | SGPL1 |
|  | ZHX2 | BUD13 | LARS1 |
|  | ZNF143 | U2AF1 | SURF6 |
|  | ZNF219 | CASP14 | BHMT |
|  | ZNF263 | MRPS23 | MSH3 |
|  | ZNF711 | HSPB1 | XRCC1 |
|  |  | DRAP1 | RPL24 |
|  |  | HSD17B12 | RFC2 |
|  |  | LCN1 | LUC7L2 |
|  |  | SPATS2 | LYAR |
|  |  | GSTM5 | DNAJC13 |
|  |  | RPS11 | BUD13 |
|  |  | SPTY2D1 | KNOP1 |
|  |  | TBL3 | RPL18 |
|  |  | BAZ2A | RPL15 |
|  |  | PLRG1 | RPL18A |
|  |  | LSG1 | GTPBP4 |
|  |  | IK | CBX8 |
|  |  | ACSL5 | PLEKHA6 |
|  |  | GNL3L | TIMM50 |
|  |  | NOC2L | CSNK1A1 |
|  |  | SERPINB4 | USP39 |
|  |  | DDX10 | POP1 |
|  |  | EIF3G | DARS1 |
|  |  | MTPAP | TFB1M |
|  |  | C1QBP | NOM1 |
|  |  | MYO1E | KAT2A |
|  |  | ZNG1A | SLC25A13 |
|  |  | TRIP12 | NOC2L |
|  |  | RPL18 | RPL37 |
|  |  | S100A11 | GNL2 |
|  |  | AIMP1 | DDX10 |
|  |  | AP2B1;AP1B1 | NAT10 |
|  |  | LYAR | OASL |
|  |  | MAP7D1 | MARK2 |
|  |  | SLC25A10 | STAU1 |
|  |  | RAD23B | ACTA1 |
|  |  | KRI1 | WDR12 |
|  |  | UBAP2L | LARP4B |
|  |  | RPL28 | CCDC47 |
|  |  | MRPS7 | MARS1 |
|  |  | LSM12 | MBD4 |
|  |  | ZC3HAV1 | TOP2A |
|  |  | MPHOSPH8 | SLC25A10 |
|  |  | RPL26L1 | RRP12 |
|  |  | EPPK1 | RPL26L1 |
|  |  | TECR | THOC2 |
|  |  | MRPS28 | KRR1 |
|  |  | C8orf33 | CHD2 |
|  |  | RPL18A | NUMA1 |
|  |  | RPL15 | MYBBP1A |
|  |  | KRR1 | MRPS22 |
|  |  | CFAP97 | SLC25A1 |
|  |  | TSR1 | DHX36 |
|  |  | PTRH1 | PHRF1 |
|  |  | WDR3 | ARPC1B |
|  |  | DDX24 | SRP68 |
|  |  | ASL | RPL9P9 |
|  |  | RPL23A | UPF3B |
|  |  | NFAT5 | DDX24 |
|  |  | NIP7 | SMARCD2 |
|  |  | PAK1IP1 | APOA1 |
|  |  | TFB1M | ABCF2 |
|  |  | LBR | SLC16A1 |
|  |  | RPL24 | DDX55 |
|  |  | MTDH | DDX31 |
|  |  | UTP18 | LARP7 |
|  |  | USP10 | MTREX |
|  |  | TPM3 | PAK1IP1 |
|  |  | CLK3 | PTDSS1 |
|  |  | CSNK1A1 | PTCD3 |
|  |  | MRPS27 | C3 |
|  |  | SBSN | TOE1 |
|  |  | IMP4 | NKRF |
|  |  | IMPDH2 | TOP2B |
|  |  | RPL9P9 | AP2B1 |
|  |  | RPL21 | IARS1 |
|  |  | LGALS7B | SRBD1 |
|  |  | CDC5L | SLC25A12 |
|  |  | DPM1 | TWNK |
|  |  | SRBD1 | RNF2 |
|  |  | HMGA2 | RFC3 |
|  |  | EXOSC10 | SAP18 |
|  |  | PARP1 | ATAD3A |
|  |  | PARP2 | NOP53 |
|  |  | SERPINB3 | PARP1 |
|  |  | MRPL16 | MRPS18B |
|  |  | EXOSC4 | SPATA5 |
|  |  | MRPS15 | ZNF48 |
|  |  | POLR1C | C7orf50 |
|  |  | SAP18 | SPTY2D1 |
|  |  | RFC1 | MTDH |
|  |  | FBXW11 | FIP1L1 |
|  |  | IMPDH1 | RARS1 |
|  |  | XPC | HSD17B12 |
|  |  | APOA1 | EPRS1 |
|  |  | RFC4 | CDC5L |
|  |  | UTP23 | EXOSC4 |
|  |  | YY1 | YTHDC2 |
|  |  | TUBB3 | BAZ1B |
|  |  | RFC5 | EXOSC7 |
|  |  | EXOSC2 | GTPBP10 |
|  |  | MYO1B | GSN |
|  |  | MAGEB2 | PNKP |
|  |  | TRMT10C | NUDT21 |
|  |  | MTHFD1L | TSR1 |
|  |  |  | CPSF1 |
|  |  |  | VIL1 |
|  |  |  | CGAS |
|  |  |  | RAI1 |
|  |  |  | POLR1C |
|  |  |  | CLK3 |
|  |  |  | MYO6 |
|  |  |  | RIOX2 |
|  |  |  | ARPC2 |
|  |  |  | TOP1 |
|  |  |  | LUC7L |
|  |  |  | CEBPZ |
|  |  |  | PHC2 |
|  |  |  | MRPS15 |
|  |  |  | BYSL |
|  |  |  | ZNF638 |
|  |  |  | PRKDC |
|  |  |  | MYO1C |
|  |  |  | ACIN1 |
|  |  |  | TEX10 |
|  |  |  | CPSF2 |
|  |  |  | MRPS27 |
|  |  |  | BOP1 |
|  |  |  | U2AF2 |
|  |  |  | ARHGEF2 |
|  |  |  | RFC5 |
|  |  |  | GNL3L |
|  |  |  | PLRG1 |
|  |  |  | TRIM56 |
|  |  |  | PWP1 |
|  |  |  | RRBP1 |
|  |  |  | MSH2 |
|  |  |  | EXOSC2 |
|  |  |  | TOR4A |
|  |  |  | EIF5B |
|  |  |  | UTP23 |
|  |  |  | STRBP |
|  |  |  | EXOSC10 |
|  |  |  | RPS6KA4 |
|  |  |  | DIMT1 |
|  |  |  | RCN2 |
|  |  |  | TRIP12 |
|  |  |  | AIMP1 |
|  |  |  | RFC4 |
|  |  |  | LBR |
|  |  |  | MYO1B |
|  |  |  | CBX4 |
|  |  |  | KARS1 |
|  |  |  | TRMT10C |
|  |  |  | U2AF1 |
|  |  |  | YY1 |
|  |  |  | HKDC1 |
|  |  |  | IMPDH2 |
|  |  |  | TCOF1 |
|  |  |  | RFC1 |
|  |  |  | BAZ2A |
|  |  |  | MYO1D |
|  |  |  | IMPDH1 |

Table S7. Correlation analysis between YY1 expression level and clinical-pathological features of Ren Ji cohort

| **Characteristics** | **YY1 expression level** | | ***p*** |
| --- | --- | --- | --- |
|  | **Low expression**  **(-and+, N=80)** | **High expression**  **(++, N=47)** |  |
| **Gender** |  |  | 1 |
| Female | 30(23.62%) | 18(14.17%) |  |
| Male | 50(39.37%) | 29(22.83%) |  |
| **Age** |  |  | 0.72 |
| <65 | 42(33.07%) | 23(18.11%) |  |
| >=65 | 38(29.92%) | 24(18.90%) |  |
| **Tumor location** |  |  | 0.2 |
| Colon | 37(29.13%) | 28(22.05%) |  |
| Rectum | 43(33.86%) | 19(14.96%) |  |
| **Grade** |  |  | 1 |
| Moderate-High | 58(45.67%) | 34(26.77%) |  |
| Low | 22(17.32%) | 13(10.24%) |  |
| **Tumor size (cm)** | |  | 0.91 |
| <5 | 52(40.94%) | 29(22.83%) |  |
| >=5 | 28(22.05%) | 18(14.17%) |  |
| **T satge** |  |  | 0.42 |
| T1 | 5(3.94%) | 1(0.79%) |  |
| T2 | 12(9.45%) | 6(4.72%) |  |
| T3 | 60(47.24%) | 40(31.50%) |  |
| T4 | 3(2.36%) | 0(0.0e+0%) |  |
| **N stage** |  |  | **0.0172** |
| N0 | 49(38.58%) | 23(18.11%) |  |
| N1 | 23(18.11%) | 10(7.87%) |  |
| N2 | 8(6.30%) | 14(11.02%) |  |
| **Distant metastasis** | |  | **0.0379** |
| M0 | 77(60.63%) | 40(31.50%) |  |
| M1 | 3(2.36%) | 7(5.51%) |  |
| **AJCC stage** |  |  | 0.12 |
| I | 14(11.02%) | 5(3.94%) |  |
| II | 35(27.56%) | 17(13.39%) |  |
| III | 28(22.05%) | 18(14.17%) |  |
| IV | 3(2.36%) | 7(5.51%) |  |
| **Tumor recurrence** |  |  | **0.0083** |
| No | 50(39.37%) | 18(14.17%) |  |
| Yes | 30(23.62%) | 29(22.83%) |  |
